# Development of a temperature-dependent chemical simulation code based on PHITS for water radiolysis from 0 to 350 °C

**DOI:** 10.1038/s41598-026-52128-z

**Published:** 2026-05-11

**Authors:** Yusuke Matsuya, Yuji Yoshii, Tamon Kusumoto, Yidi Wang, Tatsuhiko Ogawa, Tatsuhiko Sato, Takeshi Kai

**Affiliations:** 1 Faculty of Health Sciences, Hokkaido University, Sapporo, 060-0812 Japan; 2 Nuclear Science and Engineering Center, Japan Atomic Energy Agency, Tokai, 319-1195 Japan; 3 Faculty of Health Sciences, Hokkaido University of Science, Sapporo, 006-8585 Japan; 4 National Institutes for Quantum and Radiological Science and Technology, Chiba, 263-8555 Japan

**Keywords:** Water radiolysis, Temperature, G-value, Monte Carlo code, Chemistry, Materials science, Physics

## Abstract

Water radiolysis plays an important role in radiation effects on materials, including DNA damage in the human body and corrosion processes in nuclear reactors. To quantitatively evaluate radiolytic molecular yields (G-values), several Monte Carlo simulation codes for analyzing chemical species kinetics have been developed worldwide. However, conventional chemical simulation codes are generally limited to room temperature (roughly equivalent to the human body), which differs from the temperatures encountered in nuclear reactor environments. Therefore, incorporating temperature dependence into chemical simulations is essential for evaluating G-values under high-temperature conditions. In this study, we developed a chemical simulation code (PHITS-Chem) based on the general-purpose Monte Carlo code, Particle and Heavy Ion Transport code System (PHITS), applicable to the 0–350 °C temperature range. The present PHITS-Chem code explicitly accounts for the temperature dependences of diffusion coefficients and chemical reaction rate constants. The present code was benchmarked against reported experimental and theoretical G-values for low-LET (~ 0.2 keV/µm), moderate-LET (~ 11.9 keV/µm), and high-LET (~ 63.4 keV/µm) radiations, showing good agreement with the literature. The validated temperature range spans from 0 to 350 °C, covering conditions relevant to the human body, cryosphere, and light water reactors. To further improve the predictive capability and extend the applicability of the model, additional verification and updates will be required in future studies. The renewed PHITS-Chem thus enables high-precision estimation of radiolytic chemical species kinetics across a broad temperature range, which would be valuable for assessing in-core material degradation and mitigating severe accidents in nuclear reactors.

## Introduction

Ionizing radiation can induce a wide range of effects on materials through both physical and chemical processes. Especially focusing on the human body, these processes correspond to atomic interactions (such as ionizations and electronic excitations) and radiolytic chemical reactions that lead to DNA damage^1^. Among these mechanisms, the action of hydroxyl (OH) radicals, which is one of the radiolytic chemical species, can induce a high yield of DNA lesions after irradiation^2–4^. For example, 70% DNA lesions can be induced by indirect effects for low linear energy transfer (LET) radiations. For investigating such DNA damage induction mechanisms, Monte Carlo simulation codes are powerful tools^5,6^. Focusing on the chemical processes, several Monte Carlo–based chemical simulation codes, i.e., KURBUC^7^, PARTRAC^8^, TRACEL^9^, and Geant4-DNA^10^, have been developed to simulate time-dependent radiolytic yields from 1 psec to 1 µsec after energy deposition. Various types of track-structure (TS) simulation code for physical stage simulation have been developed worldwide^11^. Because of the unique features, various types of chemical codes were also developed to reproduce the experimental radiolytic chemical yields (G-values in/100 eV)^7–10^ to date. Meanwhile, water radiolysis is a key factor in radiation effects on materials, including corrosion processes in nuclear reactors^12^. Particularly, because of the difficulties associated with direct measurement of chemical yields in a reactor core, the radiolysis of water can only be evaluated with the help of computer simulations^13^. However, these conventional chemical simulation codes are generally limited to room temperature (i.e., 25 °C), which differs from the temperatures encountered in nuclear reactor environments (e.g., ~ 300°C^14,15^). Therefore, the development of chemical codes applicable to a wide range of temperatures is essential.

Among the chemical simulation codes developed worldwide, Particle and Heavy Ion Transport code System (PHITS)^16^ has recently included a dedicated step-by-step chemical simulation code, referred to as PHITS-Chem^17^. The PHITS-Chem code enables the estimation of time-dependent G-values of various chemical species under any kinds of ionizing radiations^18^ while accounting for hydroxyl (OH) radical scavenging effects. In addition, the spatial tracks of chemical species can be visualized using the PHIG-3D software^18^. To address temperature-dependent effects in water radiolysis, several chemical simulation codes have been proposed^19–21^. du Penhoat et al. and Plante developed chemical codes to calculate temperature-dependent G values in the range of 25–300 °C for light ions (^1^H^+^ and ^7^Li^3+^)^19^ and electrons (possibly applied to ^1^H^+^, ^4^He^2+^, and ^12^C^6+^)^20^, respectively; however, these codes are available only at the laboratory level (or a fee applies). More recently, the general-purpose code of the Geant4-DNA toolkit has introduced a chemical simulation framework applicable to variable temperatures, although its current applicability is limited to the range of 25–150 °C under electron exposure^21^. Compared to these codes, the PHITS-Chem code can be applicable for all radiation types^18^ thanks to the physical model named Ion Track Structure for ARbitrary Target (ITSART)^22^. Against this background, extending PHITS-Chem to predict the dynamics of chemical species over a wide temperature range (e.g., 0–350 °C) would enable the evaluation of indirect radiation effects in various environments, including those inside nuclear reactors.

Here, we developed a step-by-step radiolytic chemical simulation code, PHITS-Chem, designed for reactor-relevant applications by explicitly incorporating the temperature dependences of diffusion coefficients and chemical reaction rate constants. The code performance was benchmarked against reported experimental and theoretical G-values for low-LET (~ 0.2 keV/µm), moderate-LET (~ 11.9 keV/µm), and high-LET (~ 63.4 keV/µm) radiations over a wide temperature range from 0 to 350 °C. While existing chemical simulation codes are generally limited to near-room-temperature conditions, PHITS-Chem systematically extends the applicable temperature domain to cryogenic and high-temperature reactor environments. Finally, we will present the primary yields of various radiation types as a function of temperature. This development establishes PHITS-Chem as a versatile and robust platform for high-precision evaluation of radiolytic chemical species dynamics. It enables quantitative assessments of a wide range of phenomena, from the effects of cryogenic environments on the human body to reactor-related processes, including radionuclide production, in-core material degradation, and the mitigation of severe accidents in nuclear reactors.

## Methods

Using the PHITS-Chem code^17^ included in the PHITS package ver. 3.35^16^, we updated the code to account for temperature dependence. In this study, we newly modelled temperature-dependent diffusion coefficients for radiolytic chemical species and temperature-dependent chemical reaction rate constants. To execute the PHITS-Chem code, the track-structure (TS) mode dedicated to liquid water in PHITS was first used to output information on atomic interactions. Subsequently, using the spatial information on atomic interactions, radiolytic chemical species at 1 psec were generated based on the physicochemical model, as reported previously^18^. The generated species were then randomly diffused in liquid water using the diffusion coefficients and reacted with each other using the reaction rate constants. The simulation flow for physical, physicochemical, and chemical processes in the PHITS-Chem code is summarized in our previous report^17^. In this paper, we briefly summarize the code and introduce the new modelling related to temperature dependence.

### Physical processes

Collisions between radiation and liquid-phase water molecules are explicitly treated in several TS models implemented in PHITS (PHITS-TS)^23^: the PHITS-electron track structure (PHITS-ETS) model, the KURBUC-based ion track-structure (PHITS-KURBUC) model for protons and carbon ions, and the Ion Track Structure for ARbitrary Target (ITSART) model for all types of charged particles^22^. These models account for various atomic and molecular interaction processes, including ionizations (1b_1_, 3a_1_, 1b_2_, 2a_1_, 1a_1_), electronic excitations (A^1^B_1_, B^1^A_1_, Rydberg, diffuse bands, collective), dissociative electron attachment (DEA), molecular excitations (rotations, vibrations, phonons), and charge-exchanging processes (electron capture, electron loss). Note that the charge-exchanging processes are considered in the PHITS-KURBUC model.

In this study, benchmark simulations were performed for 1.0 MeV electrons (LET = 0.23 keV/µm), 6.2 MeV deuterons (LET = 11.9 keV/µm), and 42.8 MeV lithium ions (LET = 63.4 keV/µm) using the PHITS-ETS and ITSART models. The cut-off energies for electrons and ions were set to 1 eV and 1 keV/n, respectively. It should be noted that the electron cut-off energy must be lower than 4 eV in order to fully account for all interaction processes relevant to the generation of radiolytic chemical species (i.e., ionizations, electronic excitations, and DEA)^17^. The cross sections of molecular excitations (e.g., phonon excitations) depend on the temperature of liquid water^24^, while those of ionization and electronic excitations are independent of temperature. Considering that, it can be assumed that the physical processes are independent of temperature. However, when setting the electron cutoff energy to be lower than 1 eV, the thermalization distance of electron is affected by temperature. Thus, the cutoff energy of electron should set as 1 eV. Throughout this study, we activated the TS mode in a spherical water region, where sufficiently long radiation tracks were simulated, and we output the information on the atomic interactions using a user-defined tally. Meanwhile, in other regions, we employed the condensed-history mode of the ATIMA^25^ and electron gamma shower (EGS)^26^ models to reduce computational time.

### Physicochemical processes

In the processes, based on the branching ratio developed in our previous reports^17,18^, H_2_O^+^, H_2_O^−^, and H_2_O^*^ are immediately generated and converted into several radiolytic chemical species (e.g., H_3_O^+^, ^•^OH, e^−^ _aq_, H_2_, and H_2_O_2_). Unlike molecular excitations, the cross sections for ionization and electronic excitation are independent of temperature; therefore, temperature-independent branching ratios were used in this study. H_2_O^+^ displaced from the ionization generates two species through the proton transfer to water molecule, i.e., H_2_O^+^ + H_2_O → ^•^OH + H_3_O^+^. Meanwhile, H_2_O^*^ dissociated from the electronic excitations can produce H^•^, ^•^OH, H_2_, ^•^O^•^, and H_2_O_2_ depending on the type of electronic excitations. It should be noted that the 90% of the diffuse band excitations and 100% collective excitations of water molecules are assumed to produce H_2_O^+^ cations, which subsequently decay in the same manner as ionization, i.e., H_2_O^+^ + H_2_O → ^•^OH + H_3_O^+^, which is generally called auto-ionizations. The DEA are categorized as three types, i.e., OH^−^, O^−^, and H^−^ productions, in which ^•^OH, H_2_, and H^•^ are also subsequently generated. In addition, the electron capture and loss generate the pair of (H_3_O^+^, ^•^OH) and (H_2_O, e^−^ _aq_), respectively. It should be noted that the electron capture and loss are considered in only PHITS-KURBUC model^27^. The details are summarized in our previous report^17,18^.

Based on our previous code developments^17,18^, the spatial coordinates of radiolytic chemical species are determined as follows. Each H_2_O^+^ cation is displaced from the ionization site according to a Gaussian distribution with a mean displacement of 1.25 nm. The resulting H_3_O^+^ is assumed to be located at the same position as the H_2_O^+^, while the ^•^OH is placed at a mean distance of 0.29 nm in a random direction. For the dissociation of electronically excited water (H_2_O^*^) into H^•^ and ^•^OH, the two products are assumed to be separated by 0.87 nm along a randomly oriented line centered at the excitation site. Similarly, in the production of H_2_ and ^•^O^•^ from H_2_O^*^, the separation distance is set to be 0.58 nm. The positions of all reaction products are sampled from Gaussian distributions with standard deviations, modelled in the previous reports^17,18^. For the e^−^ _aq_, the PHITS-Chem code employs an empirical model to estimate the thermalization distance as a function of the electron cut-off energy^17,18^. The multi-step thermalization process is approximated by a single-step displacement over the mean thermalization distance in a random direction.

### Chemical processes for radiolytic species’ diffusion

The PHITS-Chem code uses the step-by-step approach for transporting the radiolytic chemical species and their chemical reaction^17,18^. The 15 types of chemical species (i.e., ^•^OH, e^−^ _aq_, H^•^, H_3_O^+^, H_2_, H_2_O_2_, HO_2_^•^, O_2_, OH^−^, O_2_^−^, HO_2_^−^, ^•^O^•^, O^−^, tris, and DMSO) diffuse randomly in water at fixed 1 psec as the step size $\tau$, with their movement governed by the diffusion coefficients *D* (m^2^/sec or cm^2^/sec), which is used for calculating the root-mean-square distance traveled ($\:\lambda$) following$\:\:\lambda\text{}\mathrm{=}\sqrt{\mathrm{6}\mathrm{D}\tau}$. The diffusion distance traveled by each species is the determined using a Gaussian distribution with an standard deviation (SD) of 10%, as reported previously^17,18^.

**Fig. 1:**
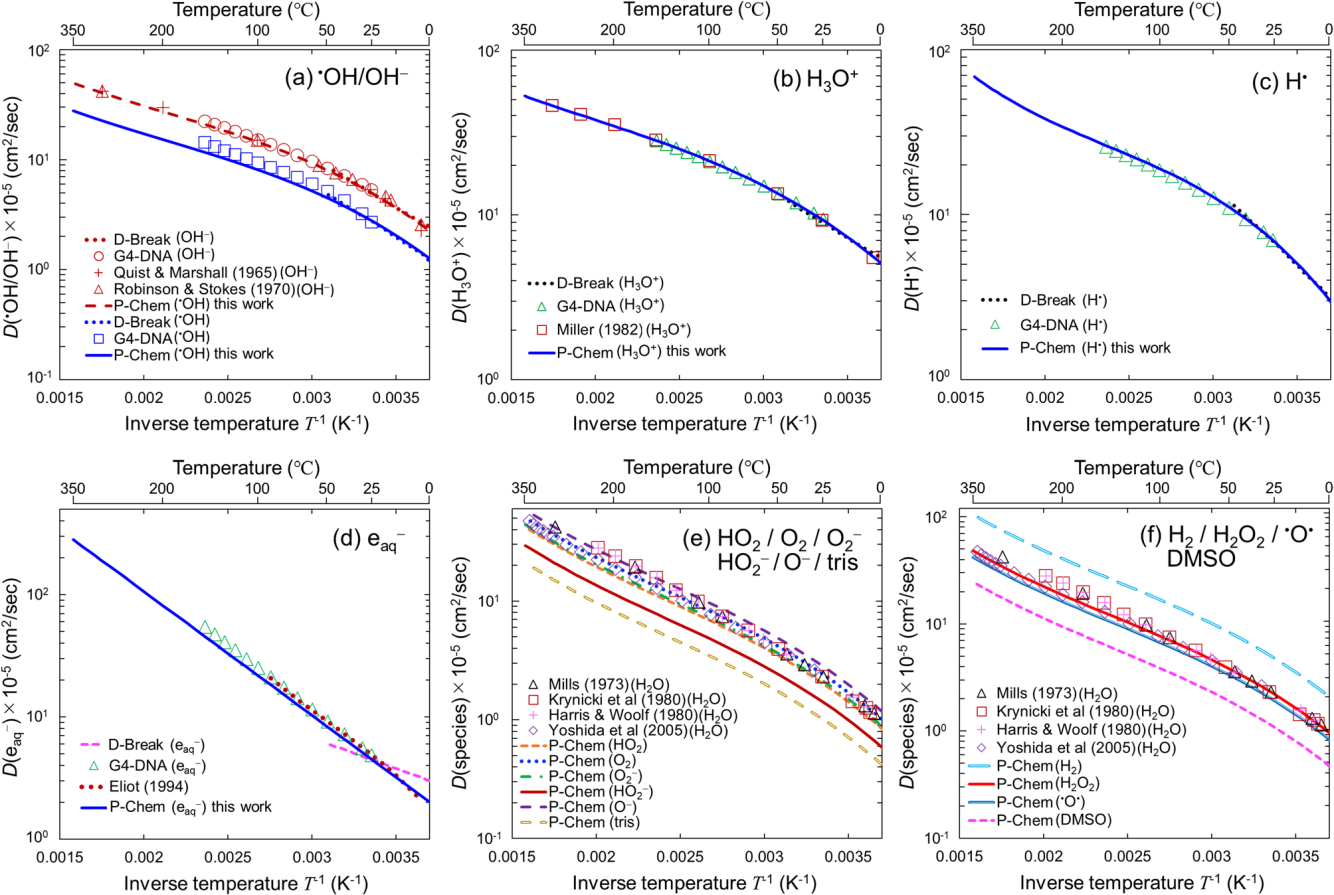
Comparison of temperature-dependent diffusion coefficients: (**a**) ^•^OH and OH^−^; (**b**) H_3_O^+^; (**c**) H^•^; (**d**) e^−^ _aq_; (**e**) HO_2_^•^, O_2_, O_2_^−^, HO_2_^−^, O^−^, tris; and (**f**) H_2_, H_2_O_2_, ^•^O^•^, DMSO. The horizontal and vertical axes are inverse of temperature in 1/K and *D* (10^–5^ cm^2^/sec), respectively. The *D* values as a function of inverse temperature (*T*^− 1^) in K^− 1 ^were compared to available literature data, including experimental results and simulation codes^21,28–35^. The model parameters for calculating the *D* values are listed in Table 1. P-Chem and G4-DNA represent the PHITS-Chem code and Geant4-DNA, respectively. It should be noted that 0.00366 (/K), 0.00335 (/K), 0.002236 (/K), and 0.00160 (/K) are 0 °C, 25 °C, 150 °C, and 350 °C, respectively.

 In our previous work, we developed the database of the diffusion coefficients *D* (m^2^/sec) for 25 °C and 1 atm. In this study, using the literature data on the temperature dependence, we newly modelled the temperature-dependent diffusion coefficient for each species. To date, the experimental values^28–35^ and the mathematical formula (i.e., polynomial function and the Arrhenius formula)^21,36^ of diffusion coefficient have been reported in several literatures. The available data in the literature is limited to OH^−^, H_3_O^+^, H^•^, e^−^ _aq_, and water (H_2_O)^21,28–35^. Considering this fact, we modelled the temperature-dependent *D* for OH^−^, H_3_O^+^, and H^•^by fitting to the literature data using a polynomial function, which is often used for expressing temperature dependence^21,37^. Note that we assumed that the temperature-dependent feature of ^•^OH is the same as that of OH^−^ due to the same molecular weight. Meanwhile, we assumed the temperature-dependent *D* of e^−^ _aq_follows the Arrhenius formula^36^, and deduced the mean activation energy (*E*_*a *_in kJ/mol) fitting to the experimental and simulation values^21,28,36^. The rest of the temperature-dependent features were obtained by fitting the polynomial function to that of water (H_2_O) reported experimentally^29–31,35^. Note that all functions were normalized using the diffusion coefficients at 25 °C so as to reproduce the chemical species yields and kinetics at 25 °C developed in our previous reports^17,18^. Fig. 1 shows the comparisons between the temperature-dependent *D *values considered in the PHITS-Chem code and the literature data^21,28–35^. Table 1 summarizes the *D* values as a function of temperature *T* in Kelvin (K), where the *D* value at 25 °C for each species is included. Using the polynomial function and Arrhenius formula (in which the model parameters are listed in Table 1), we successfully modelled the temperature-dependent *D* values for 15 types of chemical species. The developed numerical values of the *D* as a function of temperature are summarized in Table S1 of the supplementary material.

**Table 1:** Diffusion coefficients for 15 types of chemical species in the temperature range of 0 to 350 °C that are considered in the PHITS-Chem code.

Species	D (10^–9^ m^2^/s) at 25 °C	Equation (Polynomial/Arrhenius)	Reference
^•^OH	2.80	log_10_*D* = 3.05 + 1.72 × 10^3^/*T* + 5.89 × 10^5^/*T*^2^ + 9.19 × 10^7^/*T*^3^	^21,32,33^ (OH^−^)
e^−^ _aq_	4.50	*D* = exp(−*E*_*a*_/*RT*)×1.13 × 10^4^, *E*_*a*_ = 19.4 (kJ/mol), *R* = 8.31 × 10^–3^ (kJ/K/mol)	^21,28,36^ (e^−^ _aq_)
H^•^	7.00	log_10_*D* = 4.61–3.18 × 10^3^/*T* + 1.16 × 10^6^/*T*^2^−1.62 × 10^8^/*T*^3^	^21,36^ (H^•^)
H_3_O^+^	9.30	log_10_*D* = 2.67 + 9.85 × 10^2^/*T* + 3.31 × 10^5^/*T*^2^ + 5.62 × 10^7^/*T*^3^	^21,34^ (H_3_O^+^)
H_2_	5.00	log_10_*D* = 4.31 + 2.72 × 10^3^/*T* + 8.57 × 10^5^/*T*^2^ + 1.18 × 10^8^/*T*^3^	^28–35^ (H_2_O)
H_2_O_2_	2.30	log_10_*D* = 4.31 + 2.72 × 10^3^/*T* + 8.57 × 10^5^/*T*^2^ + 1.18 × 10^8^/*T*^3^	^28–35^ (H_2_O)
HO_2_^•^	2.00	log_10_*D* = 4.25 + 2.72 × 10^3^/*T* + 8.57 × 10^5^/*T*^2^ + 1.18 × 10^8^/*T*^3^	^28–35^ (H_2_O)
O_2_	2.40	log_10_*D* = 4.33 + 2.72 × 10^3^/*T* + 8.57 × 10^5^/*T*^2^ + 1.18 × 10^8^/*T*^3^	^28–35^ (H_2_O)
OH^−^	5.00	log_10_*D* = 3.31 + 1.72 × 10^2^/*T* + 5.89 × 10^5^/*T*^2^ + 9.19 × 10^7^/*T*^3^	^21,32,33^ (OH^−^)
O_2_^−^	2.10	log_10_*D* = 4.27 + 2.72 × 10^3^/*T* + 8.57 × 10^5^/*T*^2^ + 1.18 × 10^8^/*T*^3^	^28–35^ (H_2_O)
HO_2_^−^	1.40	log_10_*D* = 4.10 + 2.72 × 10^3^/*T* + 8.57 × 10^5^/*T*^2^ + 1.18 × 10^8^/*T*^3^	^28–35^ (H_2_O)
^•^O^•^	2.80	log_10_*D* = 4.25 + 2.72 × 10^3^/*T* + 8.57 × 10^5^/*T*^2^ + 1.18 × 10^8^/*T*^3^	^28–35^ (H_2_O)
O^−^	2.80	log_10_*D* = 4.40 + 2.72 × 10^3^/*T* + 8.57 × 10^5^/*T*^2^ + 1.18 × 10^8^/*T*^3^	^28–35^ (H_2_O)
tris	1.00	log_10_*D* = 3.95 + 2.72 × 10^3^/*T* + 8.57 × 10^5^/*T*^2^ + 1.18 × 10^8^/*T*^3^	^28–35^ (H_2_O)
DMSO	1.10	log_10_*D* = 3.99 + 2.72 × 10^3^/*T* + 8.57 × 10^5^/*T*^2^ + 1.18 × 10^8^/*T*^3^	^28–35^ (H_2_O)

### Chemical processes for chemical reactions

For each time step $\tau$ after diffusion (i.e., $\tau$ = 1 psec), a reaction between two radiolytic chemical species A and B was assumed to occur if their separation distance (*D*_A_+*D*_B_) was smaller than twice the reaction radius (2*a*), as reported previously^17^^18^. The reaction radius *a* is defined as *a* = *k*/4$\:\lambda$(*D*_A_+*D*_B_), where $\:\lambda$ is the root-mean-square travelled distance and *k *is the reaction rate constant. In our previous reports^17,18^, the PHITS-Chem code considers 35 types of chemical reactions (e.g., ^•^OH + ^•^OH → H_2_O_2_ and e^−^ _aq_ + e^−^ _aq_ → H_2_ + OH^−^+ OH^−^).

**Fig. 2:**
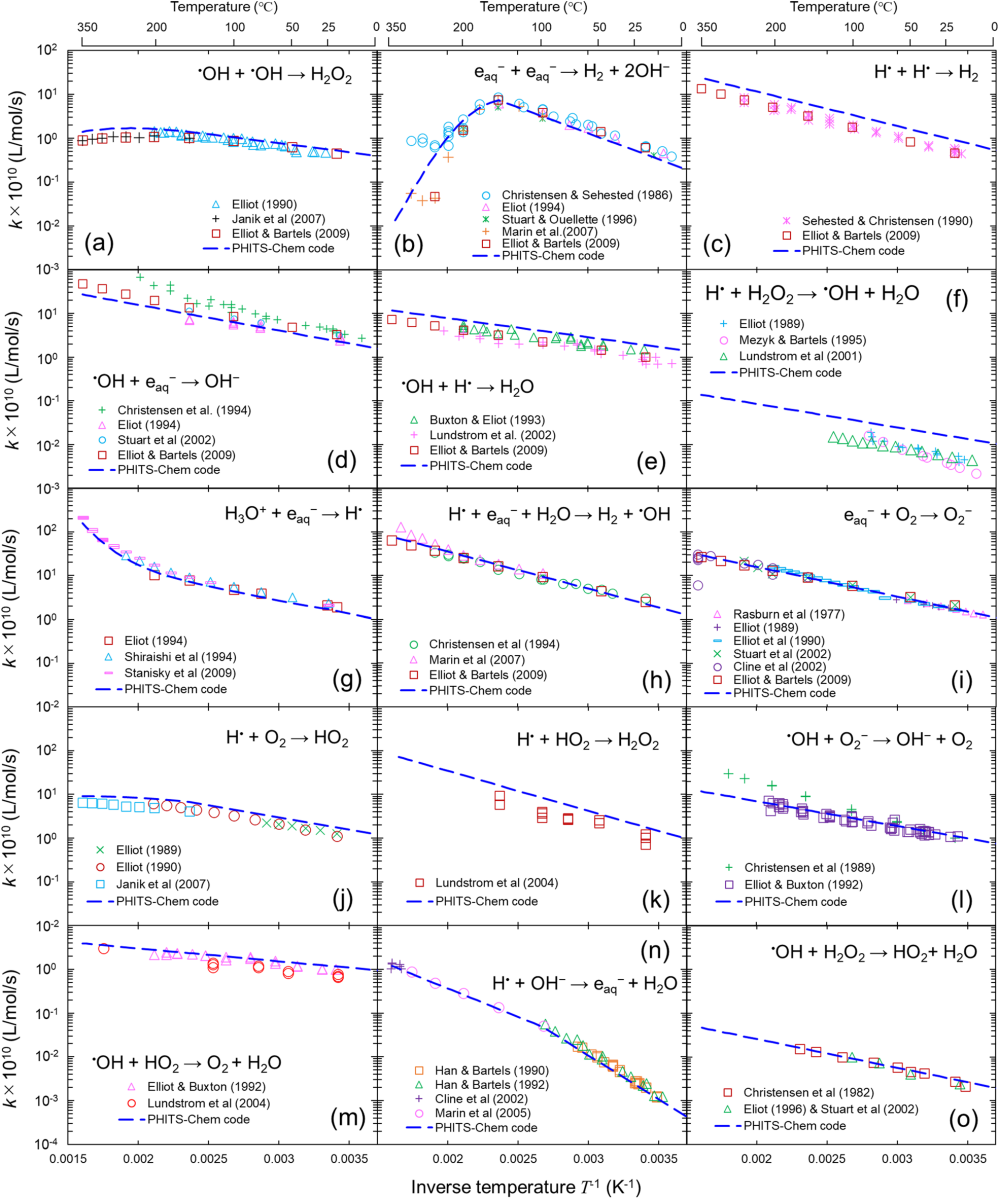
Comparison of temperature-dependent reaction rate constants: (**a**) ^•^OH + ^•^OH$\:\:\to\:\:$H_2_O; (**b**) e^−^ _aq_ + e^−^ _aq_ $\:\to\:\:$H_2_ + 2OH^−^; (**c**) H^•^ + H^•^$\:\:\to\:\:$H_2_; (**d**) ^•^OH + e^−^_aq_$\:\:\to\:\:$OH^–^; (**e**) ^•^OH + H^•^$\:\:\to\:\:$H_2_O; (**f**) H^•^ + H_2_O_2_ $\:\:\to\:\:$^•^OH; (**g**) e^−^ _aq_ + H_3_O^+^$\:\:\to\:\:$H^•^; (**h**) e^−^ _aq_ + H^•^$\:\:\to\:\:$H_2_ + ^•^OH; (**i**) e^−^ _aq_ + O_2_$\:\:\to\:\:$O_2_^–^; (**j**) H^•^ + O_2_$\:\:\to\:\:$HO_2_^•^; (**k**) H^•^ + HO_2_^•^$\:\:\to\:\:$H_2_O_2_; (**l**) ^•^OH + O_2_^–^$\:\:\to\:\:$O_2_ + OH^–^; (**m**) ^•^OH + HO_2_^•^ $\:\to\:\:$O_2_; (**n**) H^•^ + OH^–^$\:\:\to\:\:$e^−^_aq_; (**o**) ^•^OH + H_2_O_2_$\:\:\to\:\:$HO_2_^•^. The *k* values as a function of inverse temperature *T*^−1^ in K^−1 ^were compared to the available experimental data^28,38–64^. The model parameters for calculating the *k* values are listed in Table 2. It should be noted that 0.00366 (/K), 0.00335 (/K), 0.002236 (/K), and 0.00160 (/K) are 0 °C, 25 °C, 150 °C, and 350 °C, respectively.

 In the same manner as the diffusion coefficients, in this study, we newly developed the temperature-dependent *k *values using the literature data (particularly, available experimental data)^28,38–64^. We assumed that the temperature-dependent *k* values basically follow the Arrhenius formula expressed by activation energy *E*_*a*_ (kJ/mol). However, 3 types of chemical reactions (i.e., [^•^OH + ^•^OH → H_2_O_2_], [e^−^ _aq_ + e^−^ _aq_ → H_2_ + OH^−^+ OH^−^], and [H^•^ + O_2_ → HO_2_^•^]) does not follow the Arrhenius formula in the high-temperature range above 150 °C. Meanwhile, 2 types of the reactions of [e^−^ _aq_ + H_3_O^+^ → H^•^] and [H^•^ + OH^–^ → e^−^ _aq_] do not follow the Arrhenius formula in the ranges > 25 °C and > 100 °C, respectively. Therefore, for these reactions, we used the polynomial function in the same manner as the modelling of the diffusion coefficients. By fitting the formulae to the literature data, we determined the model parameters of the polynomial function and the Arrhenius formula (i.e., *E*_*a*_ values). If the literature data is unavailable, we used the *E*_*a*_= 12.6 kJ/mol in this study^65–67^. It should be noted that all functions were normalized using the diffusion coefficients at 25 °C so as to reproduce the chemical species yields and kinetics at 25 °C developed in our previous reports^17,18^, in the same manner as the modelling of the diffusion coefficients. Table 2 lists the function of the temperature-dependent *k* value for each species, in which the *k* value at 25 °C for each species is also shown. The *k* values as a function of temperature are summarized in Table S2 of the supplementary material. Using the functions, we plotted the curves of *k* values as a function of temperature, and compared the *k *values developed for the PHITS-Chem code with various literature data^28,38–64.^ Fig. 2 compares the *k* values of PHITS-Chem to the experimental data^28,38–64^ for 15 types of chemical reactions. Although the available reaction rate constants are limited to certain species, it can be confirmed that the developed rate constants were successfully determined to reproduce the experimental trends.

**Table 2:** Reaction rate constants for 35 types of chemical reactions between radiolytic chemical species in the temperature range of 0 to 350 °C that are considered in the PHITS-Chem code. The five types of chemical reactions are described using polynomial functions, with a switching temperature at which the formulation changes between the polynomial representation and the Arrhenius equation.

Radiolytic species type	Reaction ID	value (10^10^ L/mol/s) at 25 °C	Equation (Polynomial/Arrhenius)	Refs
^•^OH + ^•^OH $\:\:\to\:$ H_2_O_2_	R1	0.55	423.15 > *T* : *k* = exp(−*E*_*a*_/*RT*)×1.33 × 10^1^, *E*_*a*_ = 7.9 (kJ/mol) *T* *≥* 423.15: log_10_*k* = −5.18 + 7.73 × 10^3^/*T*−3.92 × 10^6^/*T*^2^ + 8.36 × 10^8^/*T*^3^−6.73 × 10^7^/*T*^4^	^28,48,49^
e^−^ _aq_ + e^−^ _aq_ $\:\:\:\:\to\:$ H_2_ + 2OH^–^	R2	0.50	423.15 > *T* : *k* = exp(−*E*_*a*_/*RT*)×4.55 × 10^3^, *E*_*a*_ = 22.6 (kJ/mol) *T* *≥* 423.15: log_10_*k* = 1.11 × 10^2^−2.65 × 10^5^/*T* + 2.31 × 10^8^/*T*^2^−9.47 × 10^10^/*T*^3^ + 1.86 × 10^13^/*T*^4^−1.41 × 10^15^/*T*^5^	^44–47,49^
H^•^ + H^•^ $\:\:\:\:\to\:$ H_2_	R3	1.00	*k* = exp(−*E*_*a*_/*RT*)×4.33 × 10^2^, *E*_*a*_ = 15.05 (kJ/mol)	^47^
^•^OH + e^−^_aq_ $\:\:\:\:\to\:\:$OH^–^	R4	2.50	*k* = exp(−*E*_*a*_/*RT*)×2.40 × 10^2^, *E*_*a*_ = 11.31 (kJ/mol)	^47,49^
^•^OH + H^•^ $\:\:\:\:\to\:\:$H_2_O	R5	2.00	*k* = exp(−*E*_*a*_/*RT*)×5.92 × 10^1^, *E*_*a*_ = 8.4 (kJ/mol)	^49^
H_3_O^+^ + OH^–^ $\:\:\:\:\to\:\:$H_2_O	R6	14.3	*k* = exp(−*E*_*a*_/*RT*)×9.88 × 10^2^, *E*_*a*_ = 10.5 (kJ/mol)	^65^
H^•^ + H_2_O_2_ $\:\:\:\:\to\:$^•^OH	R7	0.016	*k* = exp(−*E*_*a*_/*RT*)×1.00 × 10^0^, *E*_*a*_ = 10.26 (kJ/mol)	^54^
e^−^ _aq_ + H_3_O^+^ $\:\:\:\:\to\:\:$H^•^	R8	1.70	298.15 > *T* : *k* = exp(−*E*_*a*_/*RT*)×2.74 × 10^2^, *E*_*a*_ = 12.6 (kJ/mol) *T* *≥* 298.15: log_10_*k* = 2.90 × 10^1^+3.89 × 10^4^/*T* + 2.05 × 10^7^/*T*^2^ + 4.90 × 10^9^/*T*^3^ + 4.38 × 10^11^/*T*^4^	^28,60,61^
e^−^ _aq_ + H^•^ $\:\:\:\:\to\:\:$H_2_ + ^•^OH	R9	2.50	*k* = exp(−*E*_*a*_/*RT*)×1.79 × 10^3^, *E*_*a*_ = 16.3 (kJ/mol)	^28^
e^−^ _aq_ + O_2_ $\:\:\:\:\to\:\:$O_2_^–^	R10	1.90	*k* = exp(−*E*_*a*_/*RT*)×3.60 × 10^2^, *E*_*a*_ = 13.0 (kJ/mol)	^36^
e^−^ _aq_ + O_2_^–^ $\:\:\:\:\to\:\:$OH^–^ + HO_2_^–^	R11	1.30	*k* = exp(−*E*_*a*_/*RT*)×1.30 × 10^0^, *E*_*a*_ = 0.0 (kJ/mol)	^28,68^
e^−^ _aq_ + HO_2_ $\:\:\:\:\to\:\:$HO_2_^–^	R12	2.00	*k* = exp(−*E*_*a*_/*RT*)×4.45 × 10^2^, *E*_*a*_ = 13.4 (kJ/mol)	^68^
H^•^ + ^•^O^•^ $\:\:\:\:\to\:$^•^OH	R13	2.00	*k* = exp(−*E*_*a*_/*RT*)×3.22 × 10^2^, *E*_*a*_ = 12.6 (kJ/mol)	^66,67^
H^•^ + O_2_ $\:\:\:\:\to\:$ HO_2_^•^	R14	1.90	423.15 > *T* : *k* = exp(−*E*_*a*_/*RT*)×1.37 × 10^2^, *E*_*a*_ = 10.61 (kJ/mol) *T* *≥* 423.15: log_10_*k* = 4.12 × 10^− 1^+6.90 × 10^2^/*T*−2.18 × 10^5^/*T*^2^	^28,55^
H^•^ + O_2_^–^ $\:\:\:\:\to\:$ HO_2_^–^	R15	2.00	*k* = exp(−*E*_*a*_/*RT*)×3.22 × 10^2^, *E*_*a*_ = 12.6 (kJ/mol)	^68^
H^•^ + HO_2_^•^ $\:\:\:\:\to\:$ H_2_O_2_	R16	2.00	*k* = exp(−*E*_*a*_/*RT*)×2.33 × 10^3^, *E*_*a*_ = 17.5 (kJ/mol)	^56^
H_3_O^+^ + O_2_^–^ $\:\:\:\:\to\:\:$HO_2_	R17	3.80	*k* = exp(−*E*_*a*_/*RT*)×2.63 × 10^2^, *E*_*a*_ = 10.5 (kJ/mol)	^65^
^•^O^•^ + ^•^O^•^ $\:\:\:\:\to\:\:$O_2_	R18	2.20	*k* = exp(−*E*_*a*_/*RT*)×3.55 × 10^2^, *E*_*a*_ = 12.6 (kJ/mol)	^66,67^
^•^OH + ^•^O^•^ $\:\:\:\:\to\:\:$HO_2_	R19	2.00	*k* = exp(−*E*_*a*_/*RT*)×3.22 × 10^2^, *E*_*a*_ = 12.6 (kJ/mol)	^66,67^
^•^O^•^ + HO_2_ $\:\:\:\:\to\:\:$^•^OH + O_2_	R20	2.00	*k* = exp(−*E*_*a*_/*RT*)×3.22 × 10^2^, *E*_*a*_ = 12.6 (kJ/mol)	^66,67^
^•^OH + O_2_^–^ $\:\:\:\:\to\:\:$O_2_ + OH^–^	R21	1.20	*k* = exp(−*E*_*a*_/*RT*)×9.55 × 10^1^, *E*_*a*_ = 10.85 (kJ/mol)	^28^
^•^OH + HO_2_^•^ $\:\:\:\:\to\:$ O_2_	R22	1.20	*k* = exp(−*E*_*a*_/*RT*)×1.16 × 10^1^, *E*_*a*_ = 5.62 (kJ/mol)	^28^
^•^OH + OH^–^ $\:\:\:\:\to\:\:$O^–^	R23	1.30	*k* = exp(−*E*_*a*_/*RT*)×2.10 × 10^2^, *E*_*a*_ = 12.6 (kJ/mol)	^66,67^
H^•^ + OH^–^ $\:\:\:\:\to\:\:$e^−^ _aq_	R24	0.0021	373.15 > *T* : *k* = exp(−*E*_*a*_/*RT*)×1.17 × 10^4^, *E*_*a*_ = 38.5 (kJ/mol) *T* *≥* 373.15: log_10_*k* = 1.24 × 10^1^ −1.30 × 10^3^/*T*	^28,60,61^
^•^OH + H_2_O_2_ $\:\:\:\:\to\:\:$HO_2_^•^	R25	0.0033	*k* = exp(−*E*_*a*_/*RT*)×5.32 × 10^− 1^, *E*_*a*_ = 12.6 (kJ/mol)	^66,67^
^•^OH + O^–^ $\:\:\:\:\to\:\:$HO_2_^–^	R26	1.80	*k* = exp(−*E*_*a*_/*RT*)×2.90 × 10^2^, *E*_*a*_ = 12.6 (kJ/mol)	^66,67^
^•^OH + HO_2_^–^ $\:\:\:\:\to\:\:$HO_2_^•^ + OH^–^	R27	0.75	*k* = exp(−*E*_*a*_/*RT*)×2.31 × 10^2^, *E*_*a*_ = 14.2 (kJ/mol)	^49^
e^−^ _aq_ + HO_2_^–^ $\:\:\:\:\to\:\:$2OH^–^ + H_2_	R28	0.35	*k* = exp(−*E*_*a*_/*RT*)×1.08 × 10^2^, *E*_*a*_ = 14.2 (kJ/mol)	^49^
e^−^ _aq_ + O^–^ $\:\:\:\to\:$ 2OH^–^	R29	2.20	*k* = exp(−*E*_*a*_/*RT*)×3.55 × 10^2^, *E*_*a*_ = 12.6 (kJ/mol)	^66,67^
H^•^ + O^–^ $\:\:\:\:\to\:$ OH^–^	R30	2.00	*k* = exp(−*E*_*a*_/*RT*)×3.22 × 10^2^, *E*_*a*_ = 12.6 (kJ/mol)	^66,67^
H_3_O^+^ + O^–^ $\:\:\:\:\to\:\:$OH	R31	5.00	*k* = exp(−*E*_*a*_/*RT*)×8.06 × 10^2^, *E*_*a*_ = 12.6 (kJ/mol)	^66,67^
H_3_O^+^ + HO_2_^–^ $\:\:\:\:\to\:\:$H_2_O_2_	R32	5.00	*k* = exp(−*E*_*a*_/*RT*)×8.06 × 10^2^, *E*_*a*_ = 12.6 (kJ/mol)	^66,67^
HO_2_ + O_2_^–^ $\:\:\:\:\to\:\:$O_2_ + HO_2_^•^	R33	1.00	*k* = exp(−*E*_*a*_/*RT*)×1.61 × 10^2^, *E*_*a*_ = 12.6 (kJ/mol)	^66,67^
^•^OH + tris $\:\:\:\:\to\:$ scavenged	R34	0.15	*k* = exp(−*E*_*a*_/*RT*)×2.42 × 10^1^, *E*_*a*_ = 12.6 (kJ/mol)	^66,67^
^•^OH + DMSO $\:\:\:\:\to\:$ scavenged	R35	0.66	*k* = exp(−*E*_*a*_/*RT*)×1.06 × 10^2^, *E*_*a*_ = 12.6 (kJ/mol)	^66,67^

### Benchmark test of the renewed chemical code

We performed the benchmark testing for the developed PHITS-Chem code considering temperature-dependent diffusion coefficients and reaction rate constants by comparing the G values calculated by PHITS-Chem to available experimental values and the other simulation results. To verify the developed PHITS-Chem code, we selected the three types of charged particles, i.e., low-LET electrons with 1.0 MeV (LET = 0.23 keV/µm), 6.2 MeV deuterons (LET = 11.9 keV/µm), and 42.8 MeV Li ions (LET = 63.4 keV/µm). The sufficient length of radiation tracks (e.g., at least 2 μm) were simulated using the PHITS-ETS and ITSART models. The cutoff energies of electron and ion beams were set to be 1 eV and 1 keV/n, respectively.

First, as for the low-LET radiation, we simulated the time-dependent G values (from 1 psec to 1 µsec) of e^−^ _aq_ and the primary yields (at 1 µsec) of major products (i.e., ^•^OH, e^−^ _aq_, H_2_O_2_, and H_2_) by the 1 MeV electron beams. The simulated G values were compared to the corresponding experimental G values^47,69–80^, and the other simulation results^19–21,81^. Note that the benchmark test for the time-dependent G value focuses on only e^−^ _aq _because the picosecond pulse radiolysis (PPR) and nanosecond pulse radiolysis (NPR) data are available as the experimental results^69^. Second, we also simulated the primary yields of ^•^OH, e^−^ _aq_, H_2_O_2_, H_2_, H^•^, and H^•^+H_2_ for the deuterons and Li ions. In the same manner as the low-LET radiation, we compared the primary yields estimated by the PHITS-Chem code with the corresponding experimental G values^47^and the other simulation results^19^. From the comparison results, we evaluated the performance of the developed PHITS-Chem code.

To visually understand the temperature dependences of chemical dynamics, we depicted the kinetics of radiolytic chemical species using the PHITS’ native visualization software, PHIG-3D^82^. Note that the output data on the coordinates of radiolytic chemical species can be input in the PHIG-3D software, which enables creation of the 3D animation of the chemical dynamics later than 1 psec. In this study, we depicted the kinetics of the major products (such as ^•^OH, e^−^ _aq_, H_3_O^+^, H^•^, H_2_O_2_, and H_2_) for a 0.23 keV/µm electron, a 11.9 keV/µm deuteron, and a 63.4 keV/µm Li ion using the PHIG-3D software.

### Temperature dependence of primary yields for various ion beam irradiations

After performing the benchmark test, we predicted the temperature dependence of the primary yields (G value at 1 µsec) of ^•^OH, e^−^ _aq_, and H_2_O_2_ for various types of ion beam irradiations. Note that ^•^OH, e^−^ _aq_, and H_2_O_2_ are recognized as strong oxidizer, strong reductant, and long-lived oxidizer, respectively. We selected the 10 MeV/n ion beams of proton (^1^H^+^), deuterons (^2^H^+^), helium (^4^He^2+^), lithium (^7^Li^3+^), and beryllium (^9^Be^4+^). In the same manner as the benchmark test, the cutoff energies of ions and electrons were set to be 1 keV/n and 1 eV, respectively. The LET values for 10 MeV/n ^1^H^+^ (and ^2^H^+^), ^4^He^2+^, ^7^Li^3+^, and ^9^Be^4+^ are 4.73, 18.9, 42.5, and 75.2 keV/µm, respectively, which were calculated using the t-LET tally in the PHITS code. Note that the t-LET is the tally to obtain information on track length and dose as a function of the LET (d*E*/d*x*) of a given material. To calculate the G values using the PHITS-Chem code, the sufficient length of radiation tracks (at least 2 μm) was simulated using the PHITS-ETS and ITSART models. After the calculation, in the comparison with the temperature dependence of the primary yields of 1 MeV electron beams (LET = 0.23 keV/µm), we plotted the relationship between the inverse temperature (K^− 1^) and the primary yields (/100 eV).

## Results and Discussions

### Verification of the developed PHITS-Chem code for low-LET radiation

The PHITS-Chem code was developed to consider the temperature-dependent diffusion coefficients (*D*) and reaction rate constants (*k*), as summarized in Tables 1 and 2. The validations of the model parameters (listed in Tables 1 and 2) are shown in Figs. 1 and 2, where the temperature dependent *D* and *k *values reasonably agree with the literature data^21,28–35,38–64^. First, focusing on the low-LET radiation, we simulated the G values of 1 MeV electrons using the developed PHITS-Chem code, and compared the PHITS-Chem results to the available literature data^19–21,47,69–81^.

**Fig. 3:**
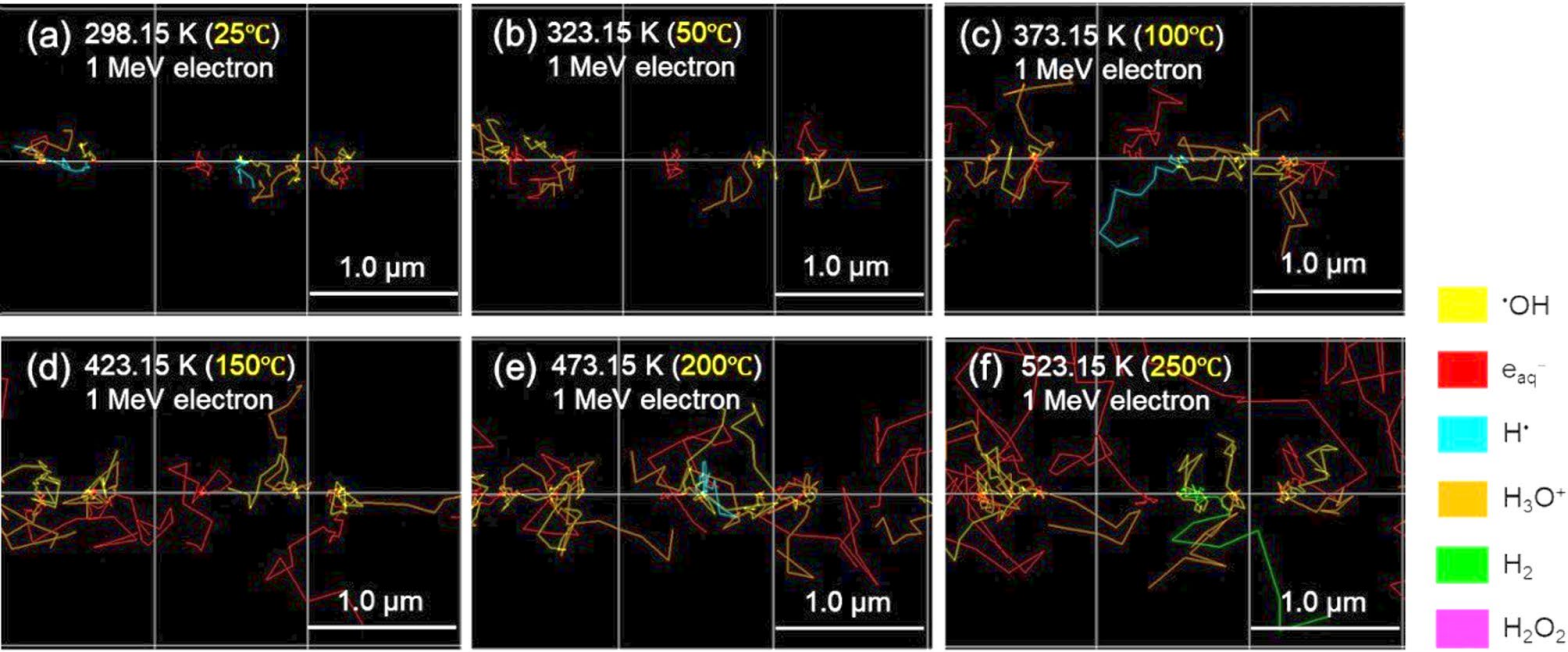
Trajectories of radiolytic chemical species generated by a 1 MeV electron: (**a**) 25 °C, (**b**) 50 °C, (**c**) 100 °C, (**d**) 150 °C, (**e**) 200 °C, and (**f**) 250 °C. We selected the major chemical products (^•^OH, e^−^ _aq_, H_3_O^+^, H^•^, H_2_O_2_, and H_2_) and made 3D illustration using the PHIG-3D software.

First, we visualized the tracks of radiolytic chemical species (^•^OH, e^−^ _aq_, H_3_O^+^, H^•^, H_2_O_2_, and H_2_) generated by 1 MeV electrons. Figure 3 shows the chemical kinetics at 25 °C (Fig. 3a), 50 °C (Fig. 3b), 100 °C (Fig. 3c), 150 °C (Fig. 3d), 200 °C (Fig. 3e), and 250 °C (Fig. 3f), as visualized using the PHIG-3D software. As illustrated in Fig. 3, although the chemical species at 25 °C diffuse at the region proximal to the electron track (which propagates from left to right along the center of the vertical axis), the species at 250 °C expand to the distal region from the track, e.g., about 1 μm at glance. This feature is natural because the *D* values monotonously increase as the temperature becomes high (see Table 1). Focusing on the minor products, H_2_ and H_2_O_2_ are rarely depicted, due to their low generation in the case of low-LET radiations.

**Fig. 4:**
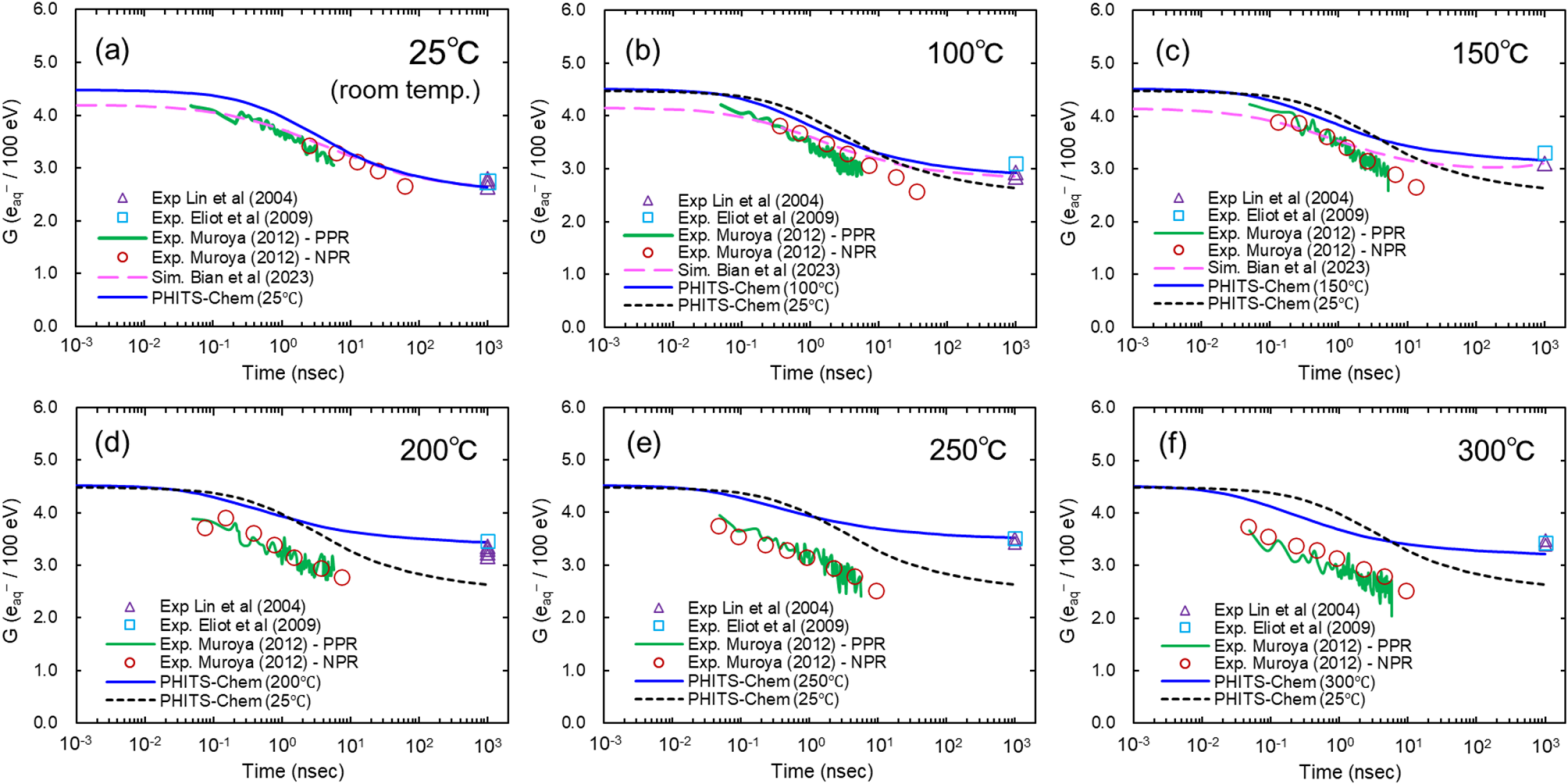
Time-dependent G value of hydrated electrons for 1 MeV electrons: (**a**) 25 °C, (**b**) 100 °C, (**c**) 150 °C, (**d**) 200 °C, (**e**) 250 °C, and (**f**) 300 °C. The predictions by the PHITS-Chem code were compared to the experimental values (including picosecond pulse radiolysis (PPR) and nanosecond pulse radiolysis (NPR))^47,69,70^and the Geant4-DNA simulation^21^.

 Among the 15 diffusion coefficients, that of the e^−^ _aq_ exhibits a linear relationship between the diffusion coefficient and the inverse temperature (1/K), as shown in Fig. 1d. This behaviour indicates that e^−^ _aq_ can diffuse the farthest among the considered chemical species, consistent with the spatial distributions shown in Fig. 3f. Figure 4 compares the time-dependent G values of e^−^ _aq_ calculated by the PHITS-Chem code to literature data, including the experimental values^47,69,70^, and Geant4-DNA simulation^21^, where the decrease tendency of e^−^ _aq_as a function of time seems to be fine. Particularly, the primary yields agree well with the experimental values^47^. Focusing on the kinetics of the G values at 200–300 °C, there are discrepancies between our simulation results and the measured data. There are several potential reasons on the discrepancies, such as dose and dose rate effects^9,81^. Both can reduce the amount of chemical species^83,84^, due to inter-track interactions. As for the dose-rate effects, recently the sparing effects of biological effects under ultra-high-dose-rate exposure have been reported^85^, which are called the FLASH effects in vivo condition^86,87^. The calculated G values are summarized in Table S3 of the supplementary material. The rest of the time-dependent G values of ^•^OH, H_2_O_2_, and H_2_ under 1 MeV electron exposure are shown in Fig. S1, where the G values were calculated for various temperature of liquid water (25–350 °C).

**Fig. 5:**
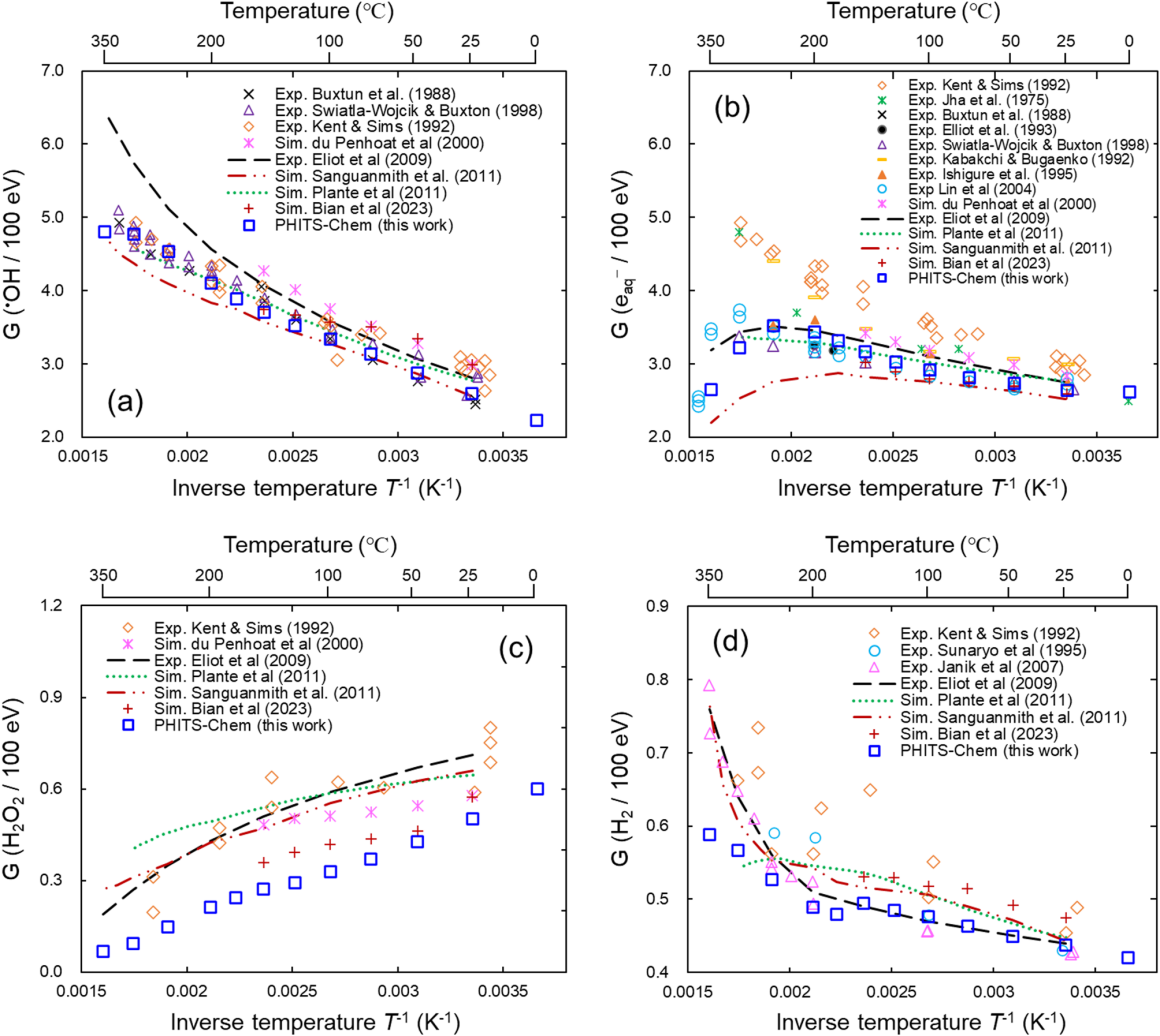
Primary yields of radiolytic chemical species for low-LET radiation: (**a**) ^•^OH, (**b**) e^−^ _aq_, (**c**) H_2_O_2_, and (**d**) H_2_. The G values at 1 µsec were calculated using the developed PHITS-Chem code, and were compared to the literature data including the experimental data^69–80 ^and the other simulations^19–21,81^. It should be noted that 0.00366 (/K), 0.00335 (/K), 0.002236 (/K), and 0.00160 (/K) are 0 °C, 25 °C, 150 °C, and 350 °C, respectively.

Considering the trends shown in Fig. 4, we calculated the primary yields of ^•^OH, e^−^ _aq_, H_2_O_2_, and H_2_, and compared the PHITS-Chem calculations to available literature data, including the experimental data^47,69–80 ^and the other simulations^19–21,81^. As shown in Fig. 5, the PHITS-Chem code could reproduce the temperature-dependent primary yields. Among 4 types of chemical species, the yields of ^•^OH, e^−^ _aq_, and H_2_ show an increase as the temperature becomes high (see Fig. 5a and b, and 5d). However, considering the increase in the reaction of [e^−^ _aq_ + H_3_O^+^$\:\:\to\:\:$H^•^] (see Fig. 2g), the primary yields of e^−^_aq_ gradually decrease as the temperature becomes high above 250 °C (0.00191 K^−1^) (see Fig. 5b). Meanwhile, the yields of H_2_ at 150–200 °C are lower than that of 150 °C. This is because the reaction rate constant of [e^−^ _aq_ + e^−^_aq_ $\:\to\:\:$H_2_ + OH^−^ + OH^−^] decreases above 150 °C (0.002236 K^− 1^) (see Fig. 2b). The phenomenon can be explained by the physical properties of water (density, dielectric constant, etc.) change with temperature. Particularly, the decrease in dielectric constant makes reactions between charged species more susceptible to Coulomb attraction and repulsion^88^. In addition, in this study, we assumed that the physical processes are independent of temperature; however, the cross sections of molecular excitations (e.g., phonon excitations) depend on the temperature of liquid water^24^. In our previous study^89^, a comparison of molecular excitation cross sections in the liquid and gas phases revealed that the cross sections in the liquid phase were significantly different from the cross sections in the gas phase without a hydrogen-bond network. Based on this, it is predicted that the formation of this network in the liquid phase becomes increasingly difficult as the temperature rises. Consequently, the cross section for phonon excitation decreases with increasing temperature, whereas that for rotational excitation increases, since the hydrogen-bond network restricts (forms) rotational (phonon) motions of water molecules. Overall, the temperature dependence of the total molecular excitation cross section is expected to be relatively limited. Based on the prediction and the agreement in Fig. 5b, this assumption of the temperature-independent physical processes seems to be reasonable approximation. However, of course, for more accurate predictions, further development on the temperature dependence of the physical processes might be necessary. Next, the yields of H_2_O_2_ monotonously decrease as the temperature becomes high (see Fig. 5c). This is because the reduction of the reaction of [^•^OH + ^•^OH$\:\:\to\:\:$H_2_O_2_]. The H_2_O_2_ yield at 25 °C (0.00335 K^−1^) is lower compared to the literature data; however, using the coefficient sets of *D* and *k *at 25 °C considered in the PHITS-Chem code, we showed the reproducibility of LET dependence as reported previously^18^. In addition, the literature data used various radiation quality categorized as low-LET radiation (e.g., 28 MeV electrons^70^ and 300 MeV protons^71^). Considering these features, the limitations, and that the gradient of decrease with increasing temperature is similar to the literature data, the coefficients included in PHITS-Chem are reasonable. However, the comparison was conducted without accounting for pressure dependence in the current version of the PHITS-Chem code. In general, within the range of high temperatures, the density (pressure) of liquid water appears to differ from 1.0 g/cm^3^. In the near future, the PHITS-Chem should be further developed to consider the pressure dependence.

### Application of developed code to moderate- and high-LET radiation

One of the advantages of the PHITS-Chem code is the account of the simulation for various charged particles^18 ^by virtue of the ITSART model^22^. Using the advantages, we next simulated the dynamics of radiolytic chemical species for moderate- and high- LET radiations, i.e., 6.2 MeV ^2^H^+^ (LET = 11.9 keV/µm) and 42.8 MeV Li ions (LET = 63.4 keV/µm), and compared the calculated primary yields to the corresponding literature data, including the simulation values^19 ^and the measured those^47^.

**Fig. 6:**
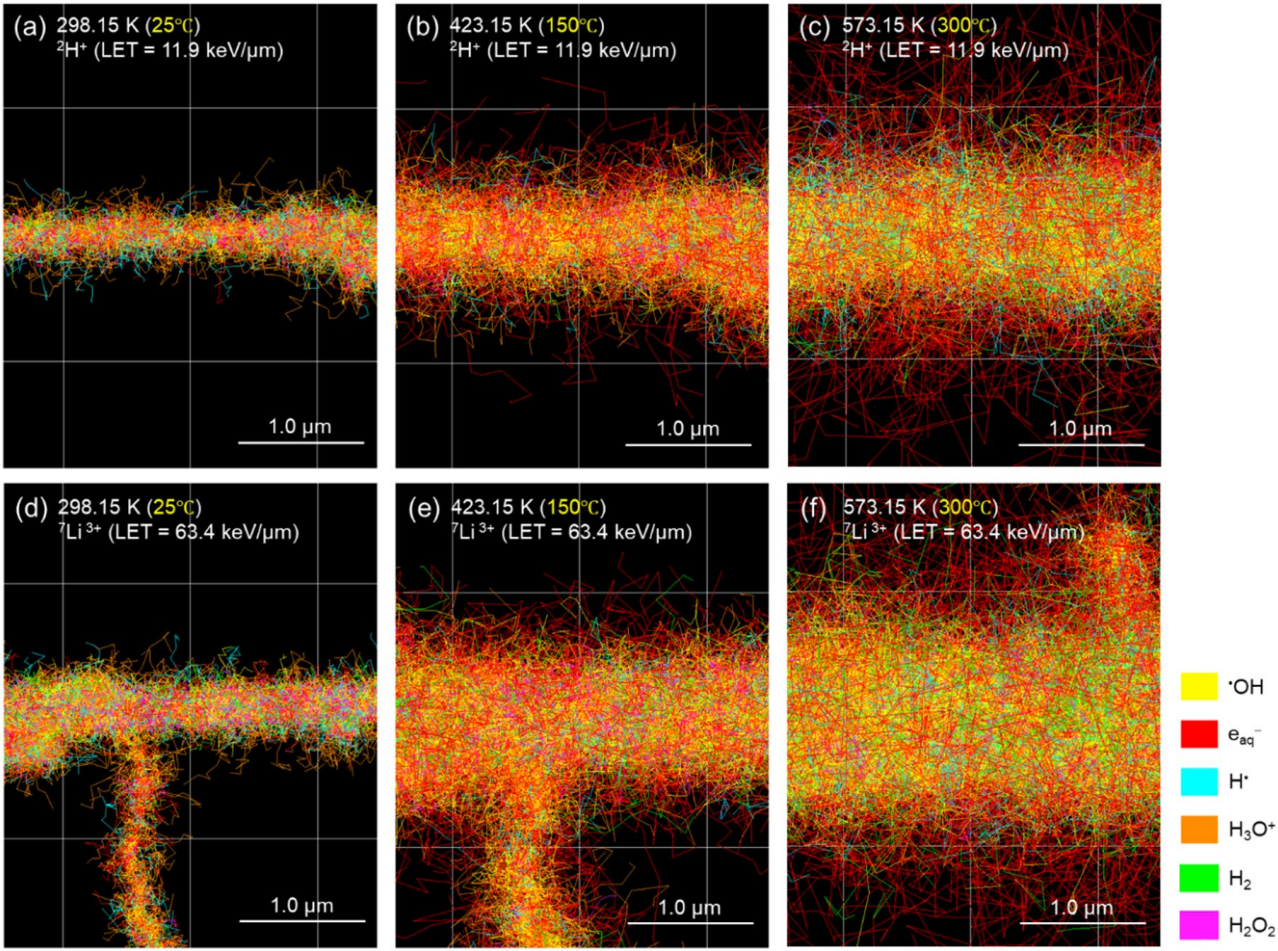
Trajectories of radiolytic chemical species generated by various ion beams: (**a**) 6.2 MeV ^2^H^+^ at 25 °C, (**b**) 6.2 MeV ^2^H^+^ at 150 °C, (**c**) 6.2 MeV ^2^H^+^ at 300 °C, (**d**) 42.8 MeV Li ions at 25 °C, (**e**) 42.8 MeV Li ions at 150 °C, and (**f**) 42.8 MeV Li ions at 300 °C. In the same manner as 1 MeV electrons, we selected the major chemical products (^•^OH, e^−^ _aq_, H_3_O^+^, H^•^, H_2_O_2_, and H_2_) and made 3D illustration using the PHIG-3D software.

**Fig. 7:**
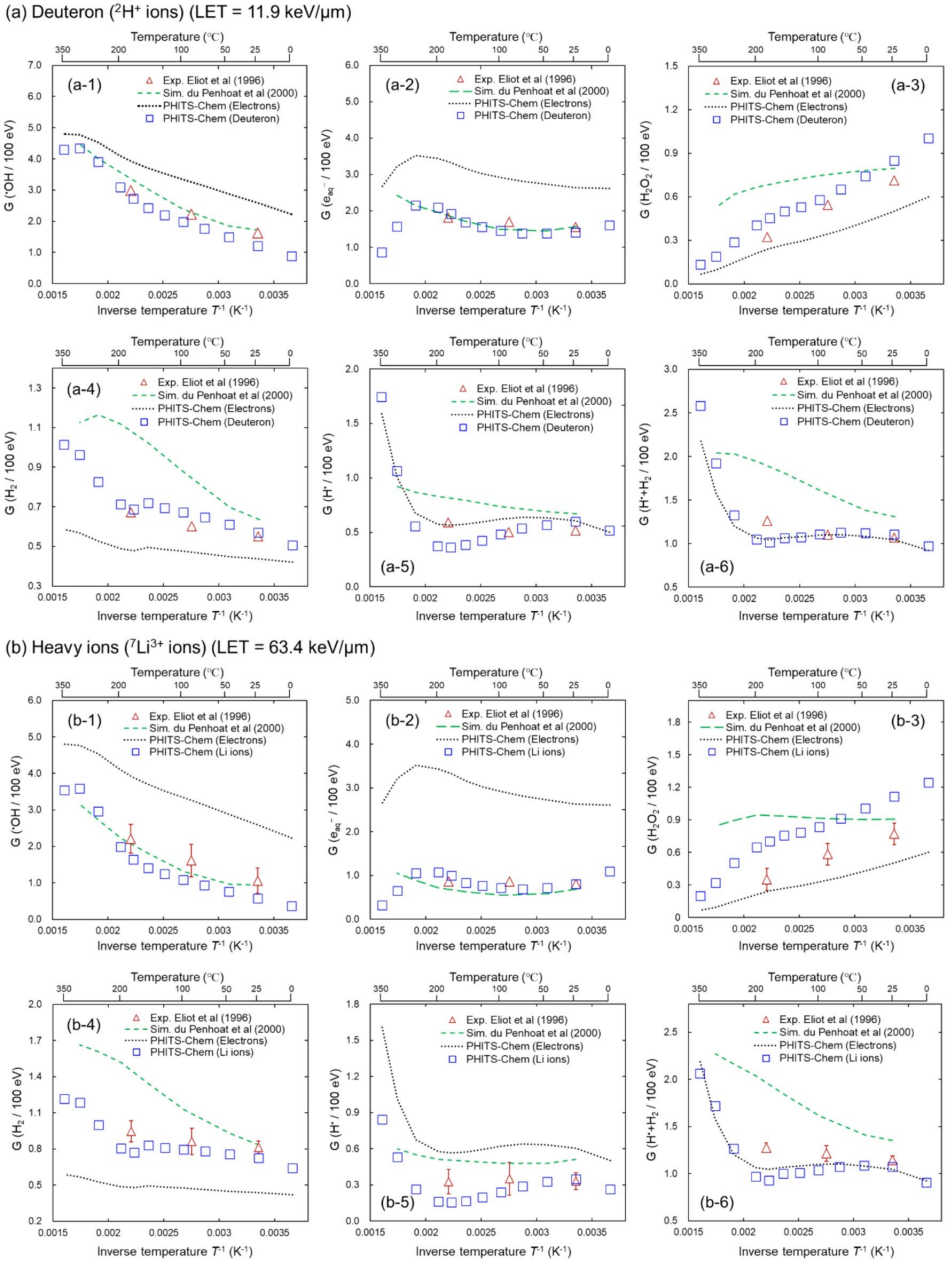
Primary yields of radiolytic chemical species for ion-beam radiations: (**a**) 6.2 MeV ^2^H^+^ and (**b**) 42.8 MeV Li ions. (a-1) and (b-1) OH radicals, (a-2) and (b-2) e^−^ _aq_, (a-3) and (b-3) H_2_O_2_, (a-4) and (b-4) H_2_, (a-5) and (b-5) H^•^, and (a-6) and (b-6) H^•^+H_2_. In the same manner as 1 MeV electrons, the G values at 1 µsec were calculated using the developed PHITS-Chem code, and were compared to the literature data (i.e., simulations^19^ and the measured those^47^. It should be noted that 0.00366 (/K), 0.00335 (/K), 0.002236 (/K), and 0.00160 (/K) are 0 °C, 25 °C, 150 °C, and 350 °C, respectively.

 Figure 6 depicts the chemical species kinetics at 25 °C (Fig. 6a and d), 150 °C (Fig. 6b and e), and 300 °C (Fig. 6c and f) visualized using the PHIG-3D software. As shown in Fig. 6, due to the increase in LET, the densities of the chemical species are higher compared to 1 MeV electron beams (see Fig. 3). Among them, a few trajectories of δ-rays can be observed in the case of high-LET Li-ion beam (see Fig. 6d and f). Figure 7 compares the PHITS-Chem calculations to available literature data^19,47^. The calculated G values for ^2^H^+^ and Li ions are summarized in Table S4 and S5, respectively. In the same manner as electron exposure (Figs. 5 and S1), the rest of the time-dependent G values of ^•^OH, H_2_O_2_, and H_2_ under the ^2^H^+^and Li ions exposure are shown in Figs. S2 and S3 where the G values were calculated for various temperature of liquid water (25–350 °C). Compared to the verifications for the low-LET radiations, the amount of available data is so limited. Despite the limited number of comparable data, we confirmed that the PHITS-Chem code can reproduce the experimental results reported by Eliot and Bartels^19^. From these comparison results, it is indicated that the simulation accuracy of the PHITS-Chem code is high; however, the available temperature range is limited to 0–350 °C. Recently, there is a paper reporting the G values for extremely high temperature of > 350°C^90,91^, showing the explosive increase in hydrated electrons^90^. In addition, the DEA cross section seems to increase as the temperature becomes higher than about 150 °C^81^. Considering these, further development of the temperature-dependent cross section of DEA, the diffusion coefficients and reaction constant rates are essential in the future study.

### Estimation of G values for various radiation types

**Fig. 8:**
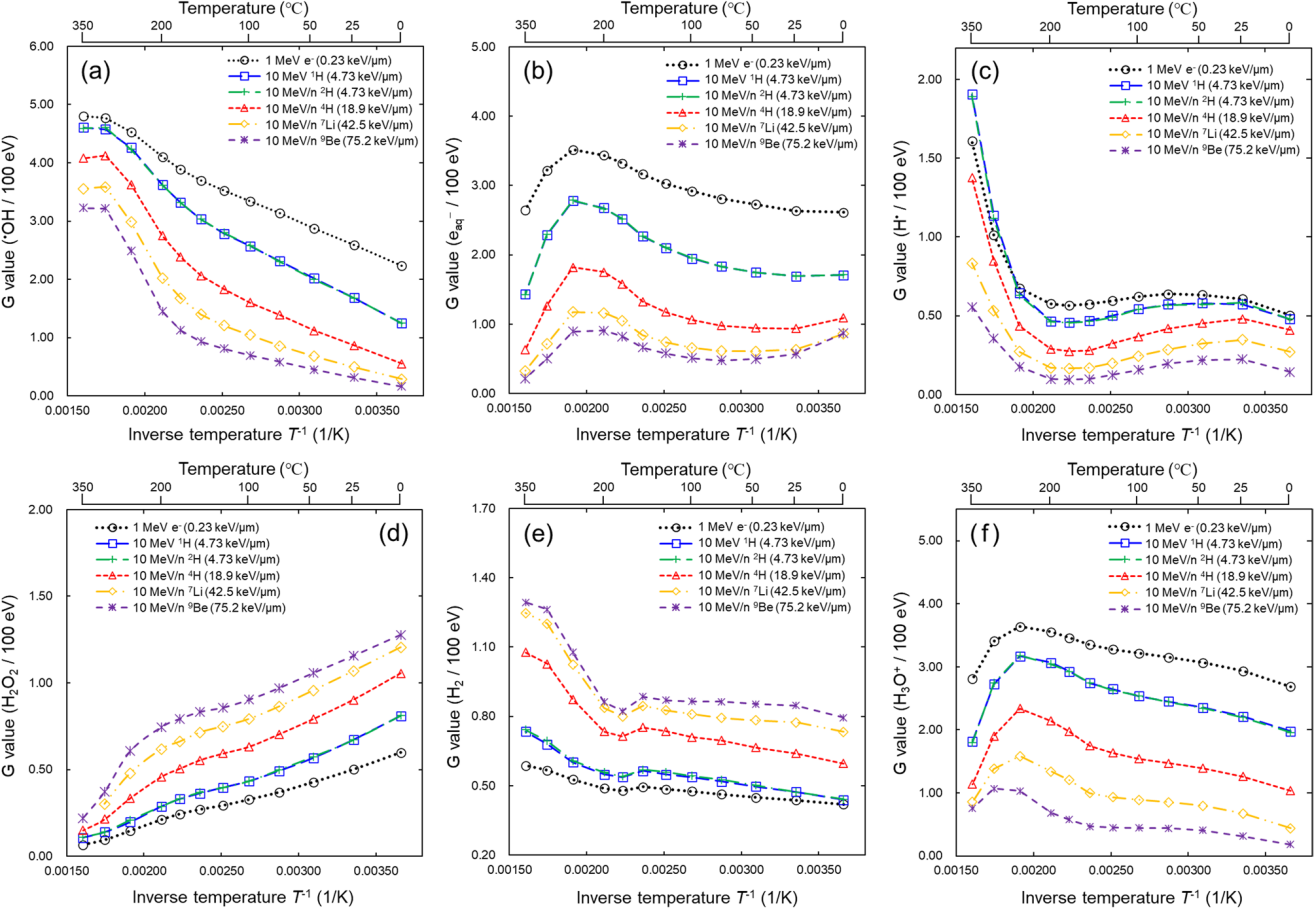
Temperature dependence of the primary yields for various types of ion beams: (**a**) ^•^OH, (**b**) e^−^ _aq_, and (**c**) H^•^, (**d**) H_2_O_2_, (**e**) H_2_, and (**f**) H_3_O^+^. Note that ^•^OH, e^−^ _aq_, and H_2_O_2_ are recognized as strong oxidizer, strong reductant, and long-lived oxidizer, respectively. We used the ITSART model as the physical model to simulate atomic interactions in liquid water. 5 types of ion beams, i.e., 10 MeV/n ^1^H^+^, ^2^H^+^, ^4^He^2+^, ^7^Li^3+^, and ^9^Be^4+^ were selected to change the LET of charged particles. It should be noted that 0.00366 (/K), 0.00335 (/K), 0.002236 (/K), and 0.00160 (/K) are 0 °C, 25 °C, 150 °C, and 350 °C, respectively.

Based on the verification, we finally predicted the primary yields as a function of inverse temperature (K^− 1^) for various types of ion beam irradiation, i.e., 10 MeV/n ^1^H^+^, ^2^H^+^, ^4^He^2+^, ^7^Li^3+^, and ^9^Be^4+^. Figure 8 depicts the temperature dependence of (a) ^•^OH, (b) e^−^ _aq_, (c) H^•^, (d) H_2_O_2_, (e) H_2_, and (f) H_3_O^+^. Among them, ^•^OH, e^−^ _aq_, and H_2_O_2_ are recognized as strong oxidizer, strong reductant, and long-lived oxidizer, respectively. As shown in Fig. 8, the yields of four radiolytic chemical species (^•^OH, e^−^ _aq_, H^•^, and H_3_O^+^) decrease with increasing LET of the ionizing radiation. Meanwhile, the yields of H_2_O_2_ and H_2_ increase with increasing LET values. Basically, the changes of primary yields of these chemical species by LET exhibits similar temperature dependences. Focusing on the G values of H_2_, the temperature dependence in the range of 25–150 °C seems to be reduced. This trend is qualitatively consistent with the recent report by Toigawa et al.^92^, where the G values of H_2 _from a plutonium solution. However, in their experimental measurements, nitric acid aqueous solutions were used, in which hydrated electrons can be scavenged during the diffusion in the solution^92^. Therefore, a direct quantitative comparison with the present results is difficult. Further improvements to the PHITS-Chem code are required to enable simulations of mixed solutions, such as nitric acid aqueous systems, in future code development.

As shown in Fig. 8, we focused on relatively light ions rather than very heavy ions (e.g., carbon, oxygen and iron ions). This choice was made because the computational cost associated with simulating the dynamics of radiolytic chemical species becomes prohibitively high for very heavy ions. In our previous study, we developed a space-partitioning method that significantly reduced the computational cost (approximately 30-fold faster in the case of electrons)^18^. However, when performing the radiolytic simulation (1 psec to 1 µsec) of carbon ion beams at 250 °C using the same geometry as Fig. 8, it takes about 6 days per track (5 tracks per month). This approach remains insufficient for heavy-ion simulations. To address this limitation, the implementation of parallel computing techniques in the PHITS-Chem code should be considered. In particular, fast calculation of radiolytic chemical species induced by α-particles is essential for future applications of PHITS-Chem to water radiolysis evaluations in nuclear reactor environments.

## Conclusions

In this study, we developed a step-by-step radiolytic chemical simulation code, PHITS-Chem, that incorporates the temperature dependences of diffusion coefficients and reaction rate constants. The developed PHITS-Chem successfully reproduces experimental and simulated primary yields for low-LET (~ 0.2 keV/µm), moderate-LET (~ 11.9 keV/µm), and high-LET (~ 63.4 keV/µm) radiations over a wide temperature range from 0 to 350 °C. These results demonstrate that the present code is applicable not only to ambient and cryogenic conditions but also to high-temperature reactor environments (~ 300 °C). The primary yields for various types of ionizing radiations are systematically evaluated using the developed framework. Overall, the PHITS-Chem code provides a reliable and versatile tool for high-precision estimation of radiolytic chemical species dynamics across a broad temperature range. Nevertheless, the present validation remains limited, particularly with respect to the temperature range investigated and the lack of consideration of temperature-dependent processes in the physical and physicochemical stages (e.g., temperature-dependent DEA and molecular excitations’ cross sections). Further investigation of these effects is necessary for more comprehensive modeling. The developed PHITS-Chem code will be made available in a future version of PHITS, after version 3.36.

## Supplementary Information

Below is the link to the electronic supplementary material.

Supplementary Material 1

## Data Availability

The data supporting this article have been included as part of the Supplementary material. The code can be obtained from the PHITS website (https://phits.jaea.go.jp/index.html) if the users submit the application form to Japan Atomic Energy Agency.
