# Supplementary material for: Development of a temperature-dependent chemical simulation code based on PHITS for water radiolysis from 0 to 350 °C
Source: Sci Rep. 2026 May 11;16:21477. doi: 10.1038/s41598-026-52128-z (PMC13350851; doi:10.1038/s41598-026-52128-z)
Supplement: Supplementary file 1 — Supplementary Material 1 [file 41598_2026_52128_MOESM1_ESM.pdf]

Supplementary Material *of "Development of a Temperature-Dependent Chemical Simulation Code based on PHITS for Water Radiolysis from 0 to 350°C"*

Table S1. Numerical values of the diffusion coefficients (10^{-9} m^2/sec) considered in the PHITS-Chem code

**°C**	**1/K**	**$^{\bullet}OH$**	**e^-_{aq}**	**$H^{\bullet}$**	**H_3O^+**	**H_2**	**H_2O_2**	**$HO_2^{\bullet}$**	**O_2**	**OH^-**	**O_2^-**	**HO_2^-**	**$^{\bullet}O^{\bullet}$**	**O^-**	**tris**	**DMSO**
0	0.00366	1.40	2.20	3.33	5.51	2.34	1.08	0.94	1.12	2.50	0.98	0.65	0.94	1.31	0.47	0.51
10	0.00353	1.91	2.97	4.69	6.96	3.29	1.51	1.31	1.58	3.42	1.38	0.92	1.31	1.84	0.66	0.72
20	0.00341	2.49	3.94	6.20	8.51	4.39	2.02	1.76	2.11	4.45	1.85	1.23	1.76	2.46	0.88	0.97
25	0.00335	2.80	4.50	7.00	9.30	5.00	2.30	2.00	2.40	5.00	2.10	1.40	2.00	2.80	1.00	1.10
30	0.00330	3.12	5.12	7.81	10.10	5.64	2.59	2.26	2.71	5.57	2.37	1.58	2.26	3.16	1.13	1.24
40	0.00319	3.79	6.55	9.47	11.73	7.01	3.22	2.80	3.36	6.76	2.94	1.96	2.80	3.92	1.40	1.54
50	0.00309	4.48	8.24	11.14	13.36	8.48	3.90	3.39	4.07	8.01	3.56	2.37	3.39	4.75	1.70	1.87
60	0.00300	5.20	10.24	12.79	14.99	10.05	4.62	4.02	4.82	9.28	4.22	2.81	4.02	5.63	2.01	2.21
70	0.00291	5.92	12.56	14.40	16.59	11.70	5.38	4.68	5.61	10.57	4.91	3.28	4.68	6.55	2.34	2.57
80	0.00283	6.65	15.22	15.97	18.16	13.42	6.18	5.37	6.44	11.87	5.64	3.76	5.37	7.52	2.68	2.95
90	0.00275	7.37	18.26	17.51	19.71	15.23	7.00	6.09	7.31	13.17	6.40	4.26	6.09	8.53	3.05	3.35
100	0.00268	8.10	21.69	19.00	21.21	17.10	7.87	6.84	8.21	14.46	7.18	4.79	6.84	9.58	3.42	3.76
110	0.00261	8.82	25.54	20.46	22.68	19.05	8.76	7.62	9.14	15.76	8.00	5.33	7.62	10.67	3.81	4.19
120	0.00254	9.55	29.82	21.90	24.11	21.07	9.69	8.43	10.12	17.05	8.85	5.90	8.43	11.80	4.21	4.64
130	0.00248	10.26	34.55	23.33	25.51	23.18	10.66	9.27	11.12	18.33	9.73	6.49	9.27	12.98	4.64	5.10
140	0.00242	10.98	39.74	24.76	26.87	25.36	11.67	10.14	12.17	19.61	10.65	7.10	10.14	14.20	5.07	5.58
150	0.00236	11.69	45.42	26.19	28.21	27.64	12.71	11.05	13.27	20.88	11.61	7.74	11.05	15.48	5.53	6.08
160	0.00231	12.41	51.58	27.63	29.51	30.00	13.80	12.00	14.40	22.15	12.60	8.40	12.00	16.80	6.00	6.60
170	0.00226	13.12	58.25	29.10	30.80	32.47	14.93	12.99	15.58	23.43	13.64	9.09	12.99	18.18	6.49	7.14
180	0.00221	13.84	65.43	30.59	32.05	35.03	16.12	14.01	16.82	24.71	14.71	9.81	14.01	19.62	7.01	7.71
190	0.00216	14.55	73.12	32.12	33.29	37.71	17.35	15.08	18.10	25.99	15.84	10.56	15.08	21.12	7.54	8.30
200	0.00211	15.27	81.33	33.69	34.51	40.50	18.63	16.20	19.44	27.28	17.01	11.34	16.20	22.68	8.10	8.91
220	0.00203	16.73	99.34	36.98	36.89	46.45	21.37	18.58	22.30	29.88	19.51	13.01	18.58	26.01	9.29	10.22
240	0.00195	18.22	119.46	40.51	39.22	52.92	24.34	21.17	25.40	32.53	22.23	14.82	21.17	29.64	10.58	11.64
260	0.00188	19.73	141.68	44.30	41.51	59.96	27.58	23.98	28.78	35.23	25.18	16.79	23.98	33.58	11.99	13.19
280	0.00181	21.28	165.96	48.40	43.77	67.61	31.10	27.05	32.45	38.00	28.40	18.93	27.05	37.86	13.52	14.87
300	0.00174	22.87	192.28	52.83	46.00	75.91	34.92	30.37	36.44	40.84	31.88	21.26	30.37	42.51	15.18	16.70
310	0.00171	23.68	206.19	55.18	47.11	80.32	36.95	32.13	38.55	42.29	33.73	22.49	32.13	44.98	16.06	17.67
320	0.00169	24.51	220.57	57.62	48.21	84.90	39.06	33.96	40.75	43.76	35.66	23.77	33.96	47.55	16.98	18.68
340	0.00163	26.18	250.77	62.79	50.41	94.62	43.53	37.85	45.42	46.76	39.74	26.49	37.85	52.99	18.92	20.82
350	0.00160	27.04	266.56	65.52	51.50	99.76	45.89	39.90	47.89	48.29	41.90	27.93	39.90	55.87	19.95	21.95

Table S2. Numerical values of the reaction rate constants (10^{10} L/mol/sec) considered in the PHITS-Chem code

℃	1/K	R1	R2	R3	R4	R5	R6	R7	R8	R9	R10	R11	R12
0	0.00366	0.411	0.217	0.574	1.65	1.47	9.70	0.0110	1.07	1.37	1.18	1.30	1.22
10	0.00353	0.465	0.308	0.725	1.96	1.67	11.43	0.0129	1.30	1.76	1.44	1.30	1.50
20	0.00341	0.521	0.428	0.902	2.31	1.89	13.30	0.0149	1.56	2.23	1.74	1.30	1.82
25	0.00335	0.550	0.500	1.000	2.50	2.00	14.30	0.0160	1.70	2.50	1.90	1.30	2.00
30	0.00330	0.580	0.581	1.105	2.70	2.11	15.33	0.0171	1.79	2.79	2.07	1.30	2.19
40	0.00319	0.641	0.774	1.338	3.11	2.35	17.52	0.0195	2.04	3.43	2.44	1.30	2.59
50	0.00309	0.704	1.012	1.599	3.56	2.60	19.84	0.0220	2.33	4.16	2.85	1.30	3.04
60	0.00300	0.769	1.303	1.892	4.04	2.86	22.31	0.0247	2.68	4.99	3.30	1.30	3.53
70	0.00291	0.835	1.653	2.217	4.55	3.12	24.92	0.0275	3.06	5.92	3.78	1.30	4.06
80	0.00283	0.903	2.068	2.574	5.09	3.39	27.66	0.0305	3.46	6.96	4.30	1.30	4.64
90	0.00275	0.973	2.557	2.964	5.66	3.67	30.52	0.0336	3.90	8.11	4.86	1.30	5.26
100	0.00268	1.044	3.124	3.388	6.25	3.95	33.50	0.0368	4.35	9.37	5.45	1.30	5.93
110	0.00261	1.115	3.779	3.845	6.88	4.24	36.60	0.0401	4.84	10.75	6.08	1.30	6.63
120	0.00254	1.188	4.526	4.336	7.53	4.54	39.80	0.0435	5.35	12.25	6.75	1.30	7.38
130	0.00248	1.261	5.373	4.861	8.20	4.83	43.10	0.0470	5.91	13.86	7.45	1.30	8.17
140	0.00242	1.335	6.325	5.419	8.90	5.14	46.49	0.0506	6.52	15.59	8.18	1.30	9.00
145	0.00239	1.373	6.843	5.710	9.26	5.29	48.22	0.0525	6.85	16.50	8.56	1.30	9.43
150	0.00236	1.410	7.389	6.010	9.62	5.44	49.97	0.0543	7.20	17.44	8.94	1.30	9.87
160	0.00231	1.467	6.578	6.634	10.36	5.75	53.54	0.0581	7.97	19.41	9.74	1.30	10.78
170	0.00226	1.519	5.558	7.290	11.12	6.06	57.18	0.0619	8.84	21.49	10.57	1.30	11.73
180	0.00221	1.564	4.479	7.977	11.90	6.37	60.89	0.0659	9.85	23.70	11.42	1.30	12.71
190	0.00216	1.603	3.460	8.696	12.70	6.69	64.67	0.0699	11.04	26.02	12.31	1.30	13.72
200	0.00211	1.635	2.576	9.445	13.51	7.00	68.50	0.0739	12.43	28.45	13.22	1.30	14.77
210	0.00207	1.659	1.859	10.223	14.34	7.32	72.39	0.0780	14.08	31.00	14.15	1.30	15.85
220	0.00203	1.677	1.307	11.030	15.19	7.64	76.33	0.0822	16.04	33.66	15.11	1.30	16.96
230	0.00199	1.688	0.901	11.865	16.04	7.95	80.32	0.0863	18.41	36.43	16.09	1.30	18.09
240	0.00195	1.692	0.612	12.726	16.91	8.27	84.35	0.0906	21.26	39.30	17.10	1.30	19.26
250	0.00191	1.689	0.411	13.614	17.79	8.59	88.41	0.0948	24.72	42.28	18.12	1.30	20.45
260	0.00188	1.681	0.275	14.527	18.68	8.91	92.50	0.0991	28.93	45.36	19.17	1.30	21.67
270	0.00184	1.667	0.184	15.464	19.58	9.22	96.63	0.1034	34.08	48.53	20.23	1.30	22.91
280	0.00181	1.648	0.123	16.424	20.48	9.54	100.77	0.1078	40.41	51.81	21.31	1.30	24.17
290	0.00178	1.625	0.083	17.407	21.40	9.85	104.94	0.1121	48.21	55.17	22.41	1.30	25.45
300	0.00174	1.597	0.056	18.411	22.32	10.17	109.13	0.1165	57.88	58.63	23.52	1.30	26.76
310	0.00171	1.565	0.039	19.435	23.24	10.48	113.33	0.1209	69.89	62.17	24.65	1.30	28.08
320	0.00169	1.531	0.027	20.480	24.18	10.79	117.55	0.1252	84.86	65.79	25.79	1.30	29.42
330	0.00166	1.493	0.019	21.542	25.11	11.10	121.77	0.1296	103.60	69.50	26.94	1.30	30.77
340	0.00163	1.454	0.014	22.623	26.05	11.40	126.00	0.1340	127.13	73.28	28.11	1.30	32.14
350	0.00160	1.412	0.010	23.721	27.00	11.71	130.23	0.1384	156.74	77.14	29.28	1.30	33.53

Table S2 (Continued)

°C	1/K	R13	R14	R15	R16	R17	R18	R19	R20	R21	R22	R23	R24
0	0.00366	1.26	1.28	1.26	1.048	2.579	1.382	1.26	1.26	0.80	0.98	0.82	0.0005
10	0.00353	1.53	1.51	1.53	1.376	3.036	1.681	1.53	1.53	0.95	1.06	0.99	0.0009
20	0.00341	1.83	1.77	1.83	1.773	3.535	2.017	1.83	1.83	1.11	1.15	1.19	0.0016
25	0.00335	2.00	1.90	2.00	2.000	3.800	2.200	2.00	2.00	1.20	1.20	1.30	0.0021
30	0.00330	2.17	2.04	2.17	2.247	4.075	2.392	2.17	2.17	1.29	1.25	1.41	0.0027
40	0.00319	2.55	2.33	2.55	2.805	4.655	2.806	2.55	2.55	1.48	1.34	1.66	0.0044
50	0.00309	2.96	2.65	2.96	3.453	5.273	3.260	2.96	2.96	1.68	1.43	1.93	0.0070
60	0.00300	3.41	2.98	3.41	4.199	5.930	3.753	3.41	3.41	1.90	1.52	2.22	0.0107
70	0.00291	3.89	3.33	3.89	5.048	6.622	4.284	3.89	3.89	2.13	1.62	2.53	0.0161
80	0.00283	4.41	3.70	4.41	6.005	7.350	4.855	4.41	4.41	2.37	1.71	2.87	0.0236
90	0.00275	4.97	4.09	4.97	7.076	8.110	5.464	4.97	4.97	2.63	1.80	3.23	0.0338
100	0.00268	5.56	4.49	5.56	8.265	8.902	6.111	5.56	5.56	2.89	1.89	3.61	0.0476
110	0.00261	6.18	4.91	6.18	9.576	9.725	6.794	6.18	6.18	3.17	1.98	4.01	0.0587
120	0.00254	6.83	5.34	6.83	11.012	10.575	7.513	6.83	6.83	3.46	2.08	4.44	0.0717
130	0.00248	7.52	5.79	7.52	12.576	11.452	8.267	7.52	7.52	3.75	2.17	4.89	0.0866
140	0.00242	8.23	6.25	8.23	14.270	12.354	9.054	8.23	8.23	4.06	2.26	5.35	0.1037
145	0.00239	8.60	6.49	8.60	15.166	12.814	9.460	8.60	8.60	4.21	2.30	5.59	0.1131
150	0.00236	8.98	6.73	8.98	16.095	13.280	9.874	8.98	8.98	4.37	2.34	5.83	0.1231
160	0.00231	9.75	7.01	9.75	18.054	14.227	10.725	9.75	9.75	4.69	2.43	6.34	0.1450
170	0.00226	10.55	7.27	10.55	20.146	15.194	11.606	10.55	10.55	5.03	2.52	6.86	0.1696
180	0.00221	11.38	7.51	11.38	22.372	16.180	12.516	11.38	11.38	5.36	2.61	7.40	0.1969
190	0.00216	12.23	7.72	12.23	24.732	17.184	13.453	12.23	12.23	5.71	2.69	7.95	0.2271
200	0.00211	13.11	7.92	13.11	27.226	18.203	14.417	13.11	13.11	6.06	2.78	8.52	0.2604
210	0.00207	14.00	8.10	14.00	29.851	19.237	15.405	14.00	14.00	6.41	2.86	9.10	0.2969
220	0.00203	14.92	8.26	14.92	32.608	20.284	16.416	14.92	14.92	6.77	2.94	9.70	0.3368
230	0.00199	15.86	8.40	15.86	35.495	21.343	17.450	15.86	15.86	7.14	3.02	10.31	0.3800
240	0.00195	16.82	8.53	16.82	38.510	22.413	18.505	16.82	16.82	7.51	3.10	10.93	0.4268
250	0.00191	17.80	8.64	17.80	41.650	23.493	19.580	17.80	17.80	7.88	3.18	11.57	0.4773
260	0.00188	18.79	8.73	18.79	44.915	24.581	20.673	18.79	18.79	8.26	3.26	12.22	0.5315
270	0.00184	19.80	8.81	19.80	48.301	25.677	21.784	19.80	19.80	8.64	3.34	12.87	0.5895
280	0.00181	20.83	8.88	20.83	51.806	26.779	22.911	20.83	20.83	9.03	3.41	13.54	0.6514
290	0.00178	21.87	8.94	21.87	55.428	27.887	24.053	21.87	21.87	9.41	3.49	14.21	0.7172
300	0.00174	22.92	8.99	22.92	59.163	29.000	25.209	22.92	22.92	9.80	3.56	14.90	0.7871
310	0.00171	23.98	9.03	23.98	63.008	30.116	26.378	23.98	23.98	10.19	3.63	15.59	0.8610
320	0.00169	25.05	9.06	25.05	66.961	31.236	27.560	25.05	25.05	10.58	3.71	16.29	0.9389
330	0.00166	26.14	9.08	26.14	71.019	32.359	28.752	26.14	26.14	10.97	3.78	16.99	1.0211
340	0.00163	27.23	9.10	27.23	75.178	33.483	29.955	27.23	27.23	11.37	3.85	17.70	1.1073
350	0.00160	28.33	9.11	28.33	79.435	34.608	31.167	28.33	28.33	11.76	3.91	18.42	1.1977

Table S2 (Continued)

℃	1/K	R25	R26	R27	R28	R29	R30	R31	R32	R33	R34	R35	R25
0	0.00366	0.0021	1.13	0.44	0.21	1.38	1.26	3.14	3.14	0.63	0.09	0.41	0.0021
10	0.00353	0.0025	1.38	0.55	0.26	1.68	1.53	3.82	3.82	0.76	0.11	0.50	0.0025
20	0.00341	0.0030	1.65	0.68	0.32	2.02	1.83	4.58	4.58	0.92	0.14	0.61	0.0030
25	0.00335	0.0033	1.80	0.75	0.35	2.20	2.00	5.00	5.00	1.00	0.15	0.66	0.0033
30	0.00330	0.0036	1.96	0.82	0.38	2.39	2.17	5.44	5.44	1.09	0.16	0.72	0.0036
40	0.00319	0.0042	2.30	0.99	0.46	2.81	2.55	6.38	6.38	1.28	0.19	0.84	0.0042
50	0.00309	0.0049	2.67	1.17	0.55	3.26	2.96	7.41	7.41	1.48	0.22	0.98	0.0049
60	0.00300	0.0056	3.07	1.37	0.64	3.75	3.41	8.53	8.53	1.71	0.26	1.13	0.0056
70	0.00291	0.0064	3.51	1.59	0.74	4.28	3.89	9.74	9.74	1.95	0.29	1.29	0.0064
80	0.00283	0.0073	3.97	1.83	0.85	4.86	4.41	11.03	11.03	2.21	0.33	1.46	0.0073
90	0.00275	0.0082	4.47	2.09	0.98	5.46	4.97	12.42	12.42	2.48	0.37	1.64	0.0082
100	0.00268	0.0092	5.00	2.37	1.11	6.11	5.56	13.89	13.89	2.78	0.42	1.83	0.0092
110	0.00261	0.0102	5.56	2.67	1.25	6.79	6.18	15.44	15.44	3.09	0.46	2.04	0.0102
120	0.00254	0.0113	6.15	2.99	1.40	7.51	6.83	17.08	17.08	3.42	0.51	2.25	0.0113
130	0.00248	0.0124	6.76	3.33	1.56	8.27	7.52	18.79	18.79	3.76	0.56	2.48	0.0124
140	0.00242	0.0136	7.41	3.69	1.72	9.05	8.23	20.58	20.58	4.12	0.62	2.72	0.0136
145	0.00239	0.0142	7.74	3.88	1.81	9.46	8.60	21.50	21.50	4.30	0.65	2.84	0.0142
150	0.00236	0.0148	8.08	4.07	1.90	9.87	8.98	22.44	22.44	4.49	0.67	2.96	0.0148
160	0.00231	0.0161	8.78	4.47	2.09	10.73	9.75	24.38	24.38	4.88	0.73	3.22	0.0161
170	0.00226	0.0174	9.50	4.89	2.28	11.61	10.55	26.38	26.38	5.28	0.79	3.48	0.0174
180	0.00221	0.0188	10.24	5.32	2.48	12.52	11.38	28.45	28.45	5.69	0.85	3.75	0.0188
190	0.00216	0.0202	11.01	5.77	2.69	13.45	12.23	30.58	30.58	6.12	0.92	4.04	0.0202
200	0.00211	0.0216	11.80	6.24	2.91	14.42	13.11	32.77	32.77	6.55	0.98	4.32	0.0216
210	0.00207	0.0231	12.60	6.72	3.14	15.40	14.00	35.01	35.01	7.00	1.05	4.62	0.0231
220	0.00203	0.0246	13.43	7.22	3.37	16.42	14.92	37.31	37.31	7.46	1.12	4.92	0.0246
230	0.00199	0.0262	14.28	7.74	3.61	17.45	15.86	39.66	39.66	7.93	1.19	5.24	0.0262
240	0.00195	0.0278	15.14	8.27	3.86	18.51	16.82	42.06	42.06	8.41	1.26	5.55	0.0278
250	0.00191	0.0294	16.02	8.81	4.11	19.58	17.80	44.50	44.50	8.90	1.33	5.87	0.0294
260	0.00188	0.0310	16.91	9.37	4.37	20.67	18.79	46.98	46.98	9.40	1.41	6.20	0.0310
270	0.00184	0.0327	17.82	9.94	4.64	21.78	19.80	49.51	49.51	9.90	1.49	6.54	0.0327
280	0.00181	0.0344	18.75	10.52	4.91	22.91	20.83	52.07	52.07	10.41	1.56	6.87	0.0344
290	0.00178	0.0361	19.68	11.11	5.18	24.05	21.87	54.67	54.67	10.93	1.64	7.22	0.0361
300	0.00174	0.0378	20.63	11.71	5.47	25.21	22.92	57.29	57.29	11.46	1.72	7.56	0.0378
310	0.00171	0.0396	21.58	12.33	5.75	26.38	23.98	59.95	59.95	11.99	1.80	7.91	0.0396
320	0.00169	0.0413	22.55	12.95	6.04	27.56	25.05	62.64	62.64	12.53	1.88	8.27	0.0413
330	0.00166	0.0431	23.52	13.58	6.34	28.75	26.14	65.35	65.35	13.07	1.96	8.63	0.0431
340	0.00163	0.0449	24.51	14.23	6.64	29.95	27.23	68.08	68.08	13.62	2.04	8.99	0.0449
350	0.00160	0.0468	25.50	14.88	6.94	31.17	28.33	70.83	70.83	14.17	2.13	9.35	0.0468

Table S3. Numerical values of the primary yields for 1 MeV electrons beam (LET = 0.23 keV/µm)

°C	1/K	$^{\bullet}OH$	e^{-}_{aq}	$H^{\bullet}$	H_3O^+	H_2	H_2O_2	$H^{\bullet}+H_2$
0	0.00366	2.230	2.615	0.504	2.686	0.420	0.599	0.924
25	0.00335	2.592	2.634	0.606	2.934	0.437	0.501	1.043
50	0.00309	2.878	2.730	0.632	3.069	0.449	0.428	1.082
75	0.00287	3.133	2.810	0.639	3.150	0.463	0.369	1.102
100	0.00268	3.342	2.918	0.621	3.218	0.476	0.328	1.097
125	0.00251	3.522	3.025	0.595	3.280	0.485	0.294	1.080
150	0.00236	3.700	3.162	0.575	3.353	0.495	0.271	1.070
175	0.00223	3.885	3.321	0.566	3.459	0.479	0.244	1.045
200	0.00211	4.101	3.435	0.576	3.553	0.489	0.212	1.064
250	0.00191	4.532	3.517	0.675	3.640	0.527	0.147	1.202
300	0.00174	4.772	3.218	1.012	3.407	0.567	0.094	1.579
350	0.00160	4.805	2.647	1.609	2.809	0.588	0.067	2.197

Table S4. Numerical values of the primary yields for 6.2 MeV $^{2}H^{+}$ (LET = 11.9 keV/µm)

°C	1/K	$^{\bullet}OH$	e^{-}_{aq}	$H^{\bullet}$	H_3O^+	H_2	H_2O_2	$H^{\bullet}+H_2$
0	0.00366	0.890	1.605	0.463	1.457	0.506	1.005	0.969
25	0.00335	1.223	1.414	0.538	1.674	0.566	0.848	1.104
50	0.00309	1.495	1.378	0.514	1.778	0.608	0.740	1.122
75	0.00287	1.769	1.396	0.479	1.861	0.645	0.644	1.124
100	0.00268	1.985	1.443	0.435	1.895	0.670	0.578	1.106
125	0.00251	2.202	1.549	0.382	1.973	0.692	0.528	1.075
150	0.00236	2.431	1.692	0.345	2.071	0.718	0.499	1.063
175	0.00223	2.723	1.920	0.329	2.261	0.683	0.453	1.012
200	0.00211	3.089	2.074	0.340	2.425	0.712	0.404	1.051
250	0.00191	3.889	2.122	0.499	2.582	0.829	0.291	1.328
300	0.00174	4.337	1.548	0.963	2.127	0.965	0.192	1.928
350	0.00160	4.293	0.858	1.557	1.317	1.020	0.134	2.577

Table S5. Numerical values of the primary yields for 42.8 MeV Li ions (LET = 63.4 keV/µm)

°C	1/K	$^{\bullet}OH$	e^{-}_{aq}	$H^{\bullet}$	H_3O^+	H_2	H_2O_2	$H^{\bullet}+H_2$
0	0.00366	0.361	1.313	0.264	0.484	0.641	1.245	0.905
25	0.00335	0.572	0.967	0.347	0.708	0.727	1.116	1.074
50	0.00309	0.755	0.869	0.326	0.829	0.758	1.006	1.084
75	0.00287	0.928	0.832	0.290	0.857	0.782	0.914	1.072
100	0.00268	1.088	0.864	0.239	0.865	0.795	0.836	1.034
125	0.00251	1.244	0.923	0.199	0.878	0.810	0.785	1.009
150	0.00236	1.404	1.008	0.167	0.900	0.832	0.757	0.998
175	0.00223	1.639	1.201	0.157	1.030	0.771	0.702	0.928
200	0.00211	1.988	1.292	0.162	1.156	0.808	0.648	0.970
250	0.00191	2.956	1.267	0.264	1.428	1.000	0.504	1.264
300	0.00174	3.583	0.778	0.530	1.335	1.186	0.320	1.716
350	0.00160	3.545	0.379	0.844	0.893	1.219	0.197	2.063

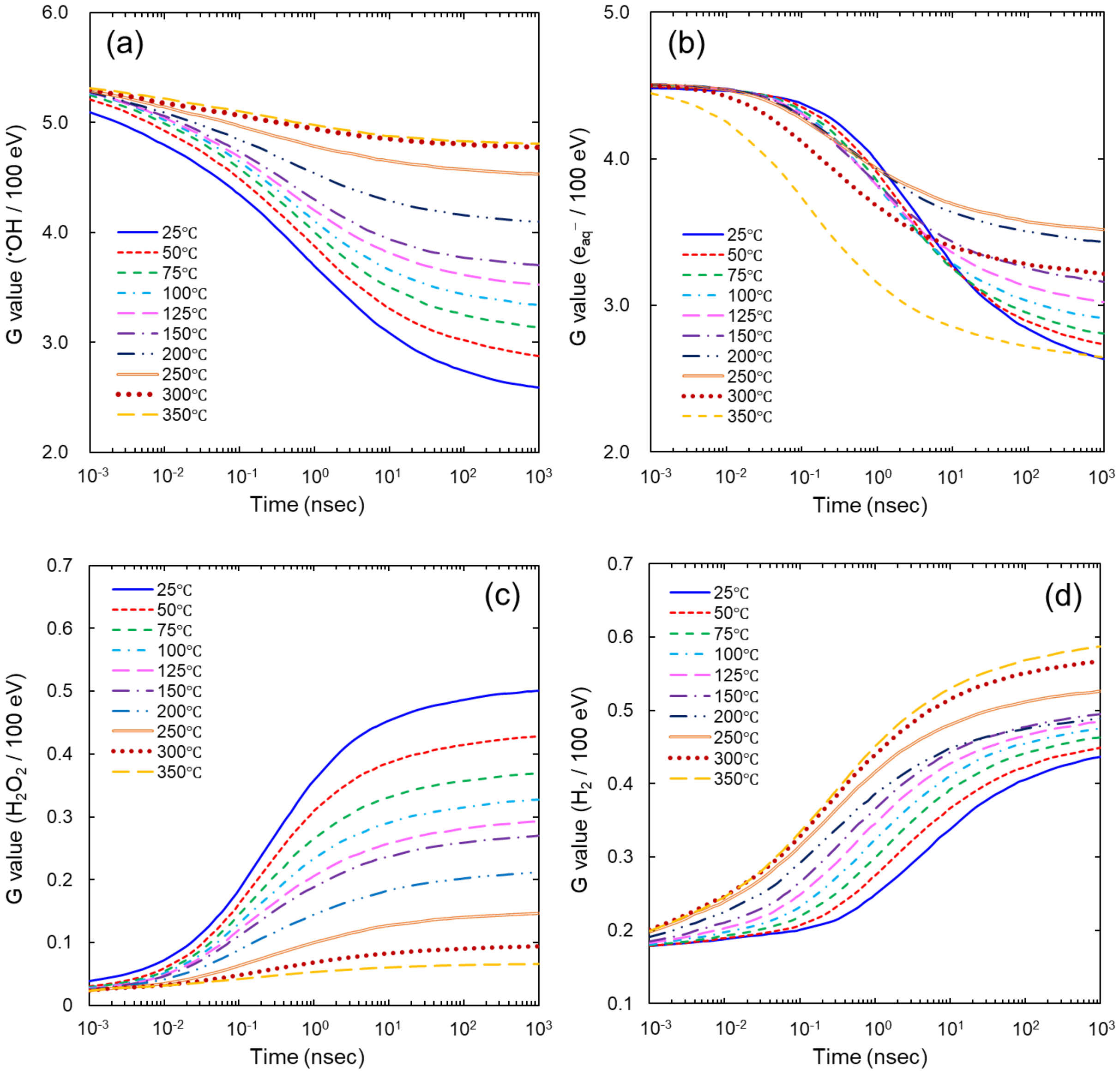

Figure S1. Time dependence of the (a) •OH, (b) e_{aq}^-, (C) H_2O_2, and (D) H_2 yields under 1 MeV electron exposure. The time-dependent G values were calculated using the PHITS-Chem code. Note that we used the PHITS-ETS model as the physical model to simulate atomic interactions of electrons in liquid water. The comparison of the primary yields between the PHITS-Chem estimation and that the literature data (corresponding experimental data and simulation results) were shown in Fig. 5 in the main text.

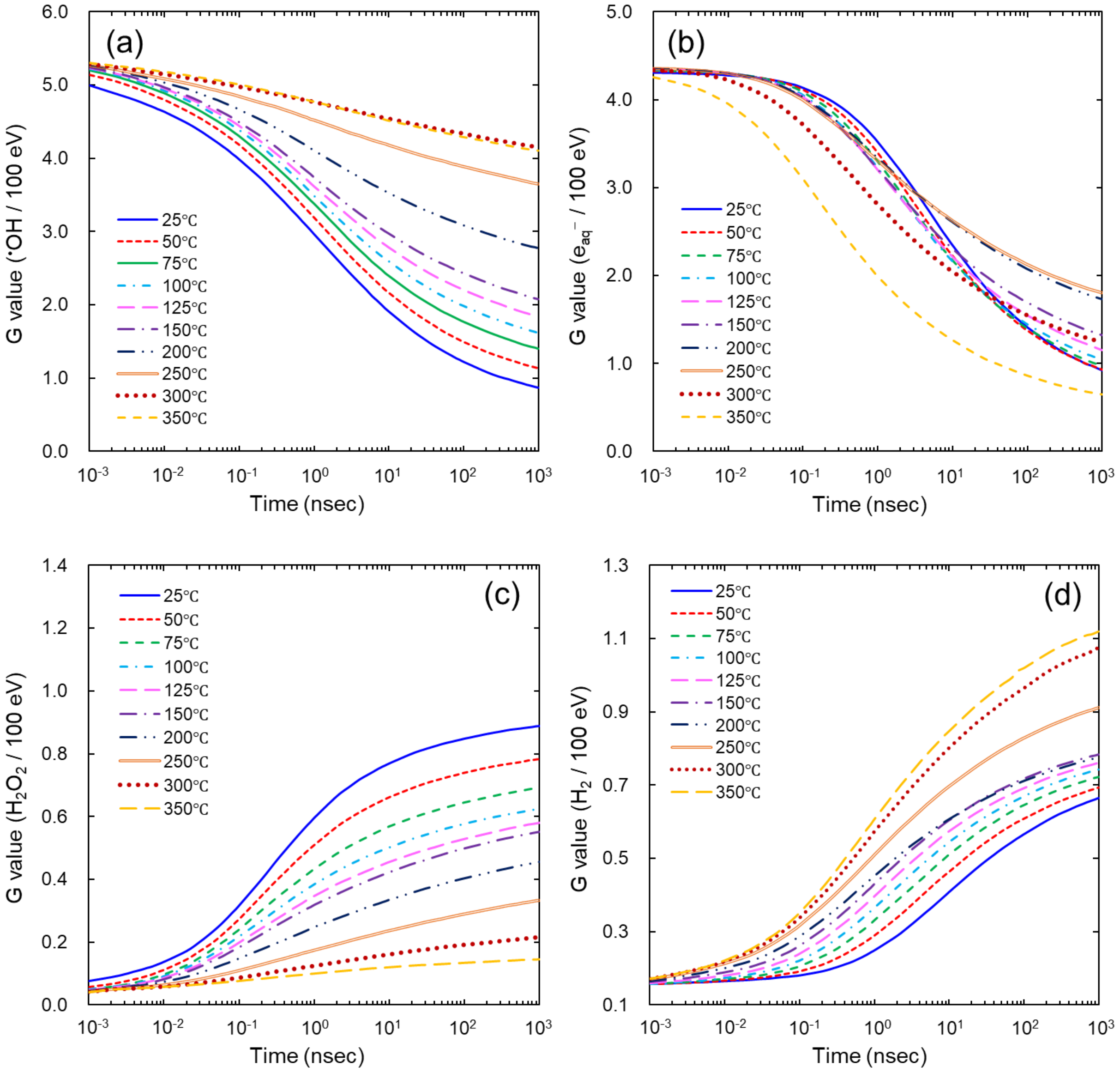

Figure S2. Time dependence of the (a) •OH, (b) e_{aq}^-, (C) H_2O_2, and (D) H_2 yields under 6.2 MeV $^2H^+$ ion exposure. The time-dependent G values were calculated using the PHITS-Chem code in the same manner as the prediction shown in Fig. S1. We used the ITSART model as the physical model to simulate atomic interactions of ion beams in liquid water. The comparison of the primary yields between the PHITS-Chem estimation and that the literature data (corresponding experimental data) were shown in Fig. 7a in the main text.

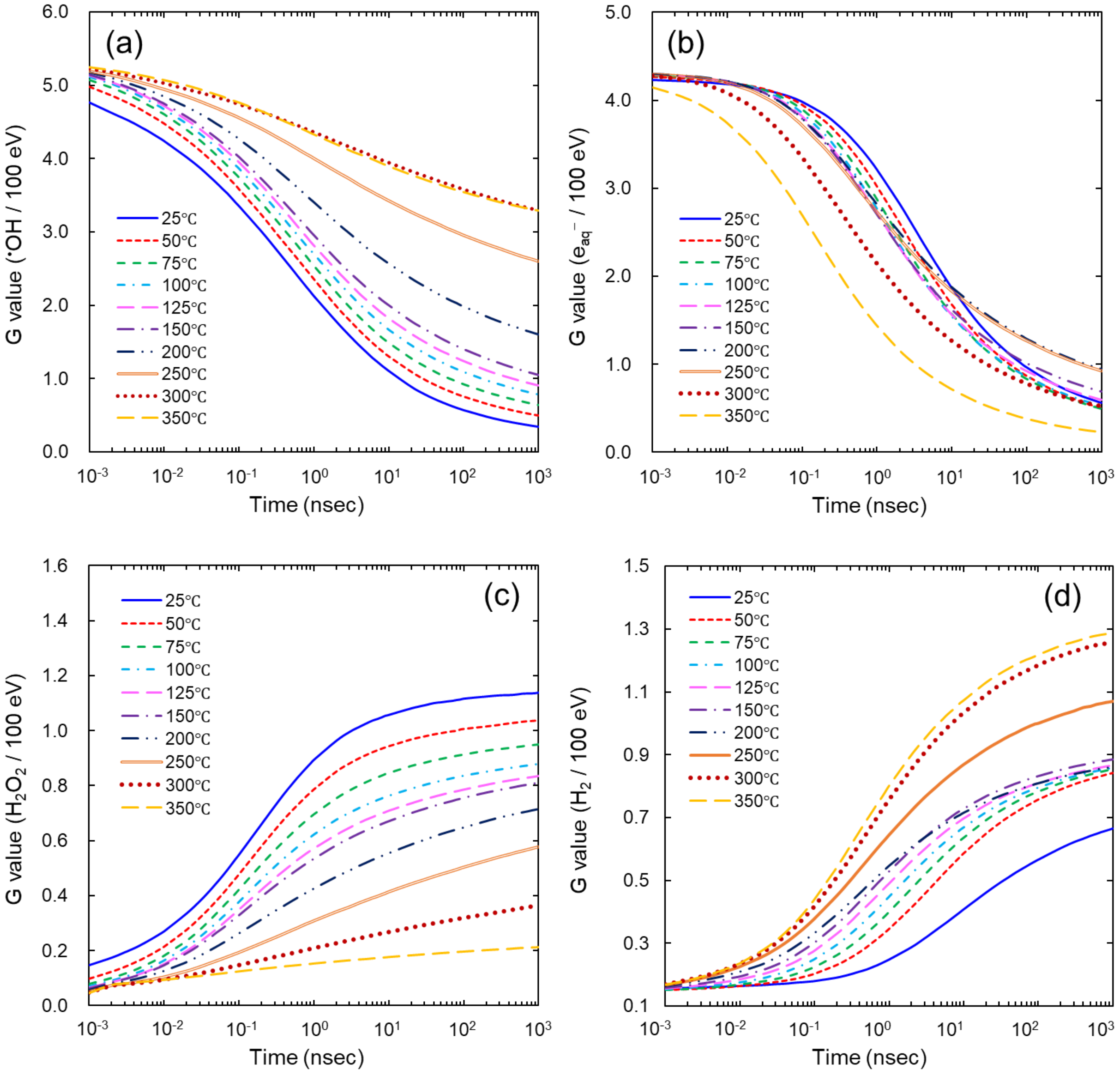

Figure S3. Time dependence of the (a) •OH, (b) e_{aq}^-, (C) H_2O_2, and (D) H_2 yields under 42.8 MeV Li ion exposure. The time-dependent G values were calculated using the PHITS-Chem code in the same manner as the prediction shown in Figs. S1 and S2. We used the ITSART model as the physical model to simulate atomic interactions of ion beams in liquid water. The comparison of the primary yields between the PHITS-Chem estimation and that the literature data (corresponding experimental data) were shown in Fig. 7b in the main text.
